# Induction of Secondary Metabolite Biosynthesis by Deleting the Histone Deacetylase HdaA in the Marine-Derived Fungus *Aspergillus terreus* RA2905

**DOI:** 10.3390/jof8101024

**Published:** 2022-09-28

**Authors:** Yao-Yao Zheng, Zhong-Lian Ma, Jing-Shuai Wu, Chang-Lun Shao, Guang-Shan Yao, Chang-Yun Wang

**Affiliations:** 1 Key Laboratory of Marine Drugs, The Ministry of Education of China, Institute of Evolution & Marine Biodiversity, School of Medicine and Pharmacy, Ocean University of China, Qingdao 266003, China; 2 Laboratory for Marine Drugs and Bioproducts, Qingdao National Laboratory for Marine Science and Technology, Qingdao 266237, China; 3 Fujian Key Laboratory on Conservation and Sustainable Utilization of Marine Biodiversity, Institute of Oceanography, Minjiang University, Fuzhou 350108, China

**Keywords:** marine-derived fungi, *Aspergillus terreus*, histone deacetylase HdaA, deletion, secondary metabolite, azaphilone

## Abstract

*Aspergillus terreus* is well-known for its ability to biosynthesize valuable pharmaceuticals as well as structurally unique secondary metabolites. However, numerous promising cryptic secondary metabolites in this strain regulated by silent gene clusters remain unidentified. In this study, to further explore the secondary metabolite potential of *A. terreus*, the essential histone deacetylase *hdaA* gene was deleted in the marine-derived *A. terreus* RA2905. The results showed that HdaA plays a vital and negative regulatory role in both conidiation and secondary metabolism. Loss of HdaA in *A. terreus* RA2905 not only resulted in the improvement in butyrolactone production, but also activated the biosynthesis of new azaphilone derivatives. After scaled fermentation, two new azaphilones, asperterilones A and B (**1** and **2**), were isolated from Δ*hdaA* mutant. The planar structures of compounds **1** and **2** were undoubtedly characterized by NMR spectroscopy and mass spectrometry analysis. Their absolute configurations were assigned by circular dichroism spectra analysis and proposed biosynthesis pathway. Compounds **1** and **2** displayed moderate anti-*Candida* activities with the MIC values ranging from 18.0 to 47.9 μM, and compound **1** exhibited significant cytotoxic activity against human breast cancer cell line MDA-MB-231. This study provides novel evidence that *hdaA* plays essential and global roles in repressing secondary metabolite gene expression in fungi, and its deletion represents an efficient strategy to mine new compounds from *A. terreus* and other available marine-derived fungi.

## 1. Introduction

In the search for new promising drug leads, filamentous fungi are playing an increasingly vital role in the production of a number of valuable secondary metabolites with great significance to human health, such as antibiotic penicillin, the cholesterol-lowering statin lovastatin, the antitumor agent paclitaxel, and the immunosuppressant cyclosporine. *Aspergillus terreus*, as an excellent producer of diverse bioactive secondary metabolites, has gained wide and intensive concerns from both microbiologists and pharmacists. The clinical drug lovastatin is industrially produced using *A. terreus* and has brought great economic value to the pharmaceutical industry [1]. It was evident that lovastatin occupied most of the market share all the time in the clinical treatment of hypercholesterolemia and other related cardiovascular diseases, with annual sales of USD 300 million [2]. Aspterric acid, a sesquiterpene derived from *A. terreus*, was reported to specifically inhibit dihydroxy acid dehydrase, which is a key enzyme in the synthesis of branched-chain amino acids in plants, with the submicromolar IC_50_ value, showing great potential for further development as a commercial herbicide [3]. Citreoviridin, an inhibitor of ATP synthase isolated from *A. terreus*, has been recently studied to treat breast cancer [4]. Additionally, a new class of azaphilones was acquired from *A. terreus* through collaborative biosynthesis, and these compounds display excellent antitumor activity demonstrated by animal experiments [5].

Histone deacetylases (HDACs) are responsible for catalyzing the removal of acetylation of the *ε* amino of histone lysine to maintain protonation, resulting in the generation of positively charged histone proteins that bind tightly to negatively charged DNA through electrostatic interactions, in turn leading to gene silencing [6]. It was found that HDACs have less of an effect on fungal growth and development and other cellular processes, and mainly inhibit the expression and synthesis of secondary metabolites [7,8]. Therefore, HDACs are ideal models for studying the relevance between histone acetylation level and secondary metabolic gene expression, and are expected to be important targets for the strain improvement by metabolic engineering.

To unearth more valuable secondary metabolites, chemical epigenetic modification and genetic manipulation strategies are widely used to activate the silent gene clusters of secondary metabolites in filamentous fungi. Chemical epigenetic modification strategy has proven to be a convenient and effective method to induce the biosynthesis of cryptic fungal metabolites [9,10,11,12,13]. Cichewicz research group systematically investigated the chemical epigenetic modification using classical HDACs inhibitors (TSA) and DNA methylase inhibitors on 12 filamentous fungi, and found that the metabolic fingerprints of 11 filamentous fungi were affected by epigenetic modifiers. As a result, novel perylenequinones were isolated from TSA-modified *Cladosporium cladosporioides* [14]. Recently, inactivation of HDACs have increasingly become a research hot spot to excavate novel fungal metabolites. For example, deletion of the HDAC gene *hdaA* resulted in the activation of 75% secondary metabolic gene clusters in *C. arbuscula*, leading to the isolation of four new compounds, including cyclic peptides arbumycin and arbumelin, diterpene arbuscullic acid A, and meroterpenoids arbuscullic acid B [15]. Knocking out *hdaA* and histone methyl transferase gene *CclA* in *Pestalotiopsis fici* resulted in the identification of 15 new compounds, including pestaloficiols T−W and 11 macrodiolide aficiolides [16]. It has been demonstrated that knockout of HDACs should be a powerful tool to tap the secondary metabolic potential of filamentous fungi.

During our consecutive investigation on secondary metabolites from marine-derived fungi [17,18,19,20,21], two new thiodiketopiperazines and ten known compounds were identified from the marine-derived fungus *A. terreus* RA2905 [22]. To explore more bioactive secondary metabolites, chemical epigenetic modification was applied to this strain by the supplement of HDAC inhibitor suberoylanilide hydroxamic acid (SAHA) in culture, resulting in the isolation of a racemate of benzyl furanone and two new benzyl pyrones [13]. By repeated manipulation with epigenetic modifiers, the metabolic profiles of *A. terreus* RA2905 were found to be changed dramatically. Although the metabolic profiles from chemical epigenetic modification have poor reproducibility, it was demonstrated that HDACs critically and negatively regulate the biosynthesis of secondary metabolites in this fungus. Inspired by the above findings, genetic manipulation aimed at HDACs was conducted to mine cryptic secondary metabolites. The critical HDAC (HdaA) was disrupted by homologous recombination, resulting in the discovery of a pair of new azaphilones with antifungal and cytotoxic activities as well as known secondary metabolites, including butyrolactones and terrein in the resultant Δ*hdaA* mutant.

## 2. Materials and Methods

### 2.1. RNA Sequencing and Bioinformatic Analysis

The conidia of *A. terreus* strains were cultured in shaking PDB liquid at 28 °C for 48 h. Total RNA was extracted from the lyophilized mycelia using TRIzol (Solarbio, Beijing, China) according to the manufacturer’s protocol for transcriptome sequencing. The quality and integrity of RNA samples were determined using Nanodrop 2100 bioanalyzer (Agilent Technologies, Palo Alto, CA, USA) and the quantity was determined with a Qubit RNA assay kit (Life Invitrogen, Waltham, MA, USA). The sequencing was performed on an Illumina Hiseq platform (Majorbio, Shanghai, China). Sequenced reads were mapped against predicted transcripts of the *A. terreus* genome using Tophat v2.0.4 (USA, available from http://tophat.cbcb.umd.edu accessed on 1 August 2022) [23]. Transcript abundance (i.e., FPKM) was estimated using the HTSeq package. AntiSMASH platform was used for bioinformatic analysis and prediction of secondary metabolites biosynthetic gene clusters.

### 2.2. General Experimental Procedure

Optical rotations for quantitative analysis of optical isomers were measured using a JASCO P-1020 digital polarimeter (589 nm, 20 °C). IR spectra for identifying functional groups were acquired on a Nicolet-Nexus-470 spectrometer with a method of KBr pellets (400−4000 cm^−1^). CD data for analysis of chiral stereoconfiguration were measured using a J-815-150S Circular Dichroism spectrometer (190−600 nm). ^1^H NMR and ^13^C NMR spectra of fractions from fungal extracts (see *Fermentation, Extraction, and Isolation* section) in deuterated methanol (methanol-*d*_4_), were recorded by JEOL JEM-ECP NMR spectrometers (500 and 125 MHz, respectively) and peaks were assigned using MestReNova software. HRESIMS spectra of compounds were tested on a Thermo MAT95XP high resolution mass spectrometer and peaks were assigned using Qual Browser Thermo Xcalibur software. The HPLC analyses of fractions from fungal extracts were performed on a HITACHI L-2000 HPLC system coupled with a L-2455 photodiode array detector and using a semi-prepared C_18_ column (Kromasil 250 × 10 mm, 5 µm). For HPLC analysis of fungal ethyl acetate extracts, the mobile phase consisted of methanol (A) and water (B) with a flowrate of 2 mL/min with 10 µL injection volume (10 mg/mL), and monitored at 190–400 nm. The elution gradient was as follows: 0–40 min, 40–100% A; 40–45 min, 100% A; 45–50 min, 100–40% A; 50–55 min, 40% A. Silica gel (Qing Dao Hai Yang Chemical Group Co., Qingdao, China; 200–300 mesh) were used for column chromatography. Precoated silica gel plates (Yan Tai Zi Fu Chemical Group Co., Yantai, China; G60, F-254) were used for thin-layer chromatography analysis.

### 2.3. Fungal Cultures

The fungus *A. terreus* RA2905 was isolated from the sea hare *A. pulmonica* (GXWZ-29) collected from the Weizhou coral reefs in the South China Sea in April 2010, and stored in China Center for Type Culture Collection (CCTCC AF 2021052), Wuhan, China. This fungal strain was identified as *A. terreus* according to its morphological traits and ITS sequence with the GenBank (NCBI) accession number MK 611650, as described previously [13].

### 2.4. Construction and Verification of A. terreus RA2905 ΔhdaA Mutant

Taking the *hdaA* (AN8042) sequence of *Aspergillus nidulans* as an inquiry, the *hdaA* gene of the strain *A. terreus* RA2905 was located and identified in the genomic sequence via Local-BLAST search, revealing a sequence of 760 amino acids with 72.6% identity to the query. Subsequently, DNA fragments were constructed through polymerase chain reactions (PCRs) which were performed using Fast-Pfu DNA polymerase (Transgen, Beijing, China). Primers were designed (Primer premier software) to amplify the 1.5 kb upstream (F1: 5′-CTGAAGGGCGGTGCCAAGAA-3′; 9818hphR: 5′-GCTCCTTCAATATCAGTTAACGTCGTGGATATGTCAAACGGAGAG-3′) and the 1.5 kb downstream homologous sequence of the *hdaA* coding sequence (9818hphF: 5′-aaattccgtcaccagccctgggttgGTAAGGGCAAGGTTGTTGCA-3′; R1: 5′-TCGAGGGACAGGCGTTCTAT-3′) from the genomic DNA of the strain *A. terreus* RA2905. The hygromycin B resistance gene as the selectable marker was connected to the location between the upstream and downstream by the fusion PCR method [24] to compose the deletion cassette (Detailed procedures of PCR, see Figure S19). Deletion of the *hdaA* gene in the WT strain was performed through homologous recombination using the protoplast transformation method as described in [25]. A 400 mg/L hygromycin B-supplemented medium was used for the screening of the transformant. Additionally, the genomic DNA of the positive selected transformant was further verified through diagnostic PCR. The 2.1 kb fragments could be amplified from the correct ∆*hdaA* mutant using the primers F1-R1 (F1: 5′-CTGAAGGGCGGTGCCAAGAA-3′, R1:5′-TCGAGGGACAGGCGTTCTAT-3′), while a 3.3 kb product could be obtained using the genomic DNA of the WT. Then, the amplified fragments were further digested by restriction enzyme *EcoR* I (Takara Bio Inc., San Jose, CA, USA), and 1.5 and 1.8 kb products could be obtained for the WT, while the correct transformant could harvest 1.4 and 0.7 kb fragments.

### 2.5. Fermentation, Extraction, and Isolation

The ∆*hdaA* mutant strain of *A. terreus* was cultivated in the potato dextrose broth (Hopebio, Qingdao, China) medium with twenty 500 mL Erlenmeyer flasks (200 mL each) at 28 °C for 7 days according to the directions. The fermentation broth was extracted repeatedly with an equal volume of ethyl acetate (EtOAc) for three times, and the organic solvent was evaporated to dryness under vacuum to give a crude extract (1.8 g). The extract was separated by silica gel column chromatography (CC) using a step gradient elution with petroleum ether/EtOAc (10:1 to 1:10, *v*/*v*) to provide five fractions (Fr.1-Fr.5). Fr.3 was further subjected to reversed phase C_18_ silica gel CC with 60% MeOH in H_2_O to obtain compounds asperterilone A (18 mg) (hereafter named as compound **1**) and asperterilone B (15 mg) (hereafter named as compound **2**) used for NMR, HRESIMS, IR, and ECD experiments.

### 2.6. Biological Assay

The minimum inhibitory concentrations (MICs) were determined using the broth dilution method as described previously [26]. Three pathogenic *Candida* species, *C. albicans* ATCC 24433, *C. parapsilosis* ATCC 22019, and *C. tropicalis* ATCC 20962, were used for anti-*Candida* activity testing. The three pathogenic *Candida* strains were cultivated in yeast extract peptone dextrose (YPD) culture medium (Sangon Biotech, Shanghai, China) at 28 °C for 72 h. The YPD seed culture with an inoculum of 10^6^ colony forming units was diluted 100 times with RPMI-1640 broth (Sangon Biotech, Shanghai, China) and delivered into sterile 96-well plates. Compounds to be tested were dissolved in DMSO at the same concentrations of 12 mg/mL. Then, the tested compounds were diluted with broth medium in the microplate wells by serial 2-fold dilutions of compounds over the range of 1−256 μg/mL (with the final test volume of 200 μL broth in each well) in triplicate for the MIC assay. The culture plates were incubated at 28 °C for 24 h. The MIC was defined as the lowest concentration that prevented the visible growth of *Candida* strains. Amphotericin was used as the positive control.

The cytotoxic activity of compounds **1** and **2** toward human breast cancer cell line MDA-MB-231 was determined in 96-well plates by the SRB method [27]. Briefly, exponentially growing MDA-MB-231 cells were harvested and plated in 96-cell plates (4 × 10^3^ cells per well). After 24 h incubation, compounds **1** and **2** with a concentration of 10 μM were added to each well and further incubated for 48 h. The cultures were fixed with ice-cold 50% trichloroacetic acid and the fixed cells were stained with 0.4% SRB (Sigma Life Science, Burlington, MA, USA). The absorbance of SRB solution was measured at 515 nm. Cisplatin was used as a positive control. The experiments were carried out in triplicate.

## 3. Results

### 3.1. Deletion of the Major HDAC in A. terreus RA2905

**Figure 1:**
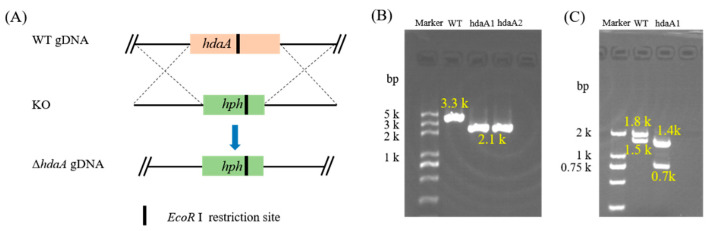
(**A**) Knockout of histone deacetylase gene *hdaA*. WT gDNA: The genome of wild-type strain; KO: Knock out; Δ*hdaA* gDNA: The genome of Δ*hdaA* mutant strain. (**B**) Diagnostic PCR amplification. (**C**) Restriction analysis of PCR products of WT and ∆*hdaA* strains.

Genome sequencing and antiSMASH analysis revealed that the genome of *A. terreus* RA2905 contains 64 secondary metabolite biosynthetic gene clusters covering diverse types of metabolites encoded by related biosynthetic enzymes. Among these gene clusters, 18 of those are predicted to encode polyketides. However, most of those compounds remain undetected in our previous analysis [13], suggesting that most of these gene clusters may silence or lowly express under these cultures. To overcome the poor reproducibility of chemical epigenetic modification, we attempted to disrupt the major HDAC (HdaA) to explore the fungal metabolic diversity. Genome analysis demonstrated that a total of 8 genes were annotated as HDAC in *A. terreus* RA2905 (Table 1). RNA sequencing analysis revealed that *gene9818* had higher expression levels compared to the other seven putative HDAC-encoding genes under PDB culture condition. This can suggest that *gene9818* might have a critical regulatory role in the expression of secondary metabolite gene clusters under the given growth conditions, compared to the other putative HDAC-encoding genes identified in this study. BLAST analysis revealed that the HDAC in *A. terreus* RA2905 encoded by *gene9818* was the homolog of HdaA, showing 100%, 75.4%, 75.6%, and 72.6% sequence identity in *A. terreus* isolated from different growing environments (e.g., NIH 2624 and ATCC 20542), *A. niger*, *A. oryzae*, and *A. nidulans*, respectively, suggesting that the HDAC-encoding genes can be conserved among different *Aspergillus* species. The elevated expression levels of *gene9818* compared to the other putative histone deacetylase-encoding genes identified in our *A. terreus* strain, and the homology (>70%) of *gene9818* with the *hdaA* gene encoded by different members of the *Aspergillus* taxon, prompted us to select *gene9818* as a candidate for studying its role in the production of secondary metabolites. The homologous recombination-based method was adopted to delete *gene9818* (referred to as *hdaA* hereafter), with the hygromycin B resistance gene (*hph*) as the selection marker. After screening on the selection media, a total of 11 hygromycin B-resistant transformants were obtained. Diagnostic PCR analysis (Figure 1) confirmed that two of these transformants showed complete removal of the *hdaA* gene, and after several generations of subcultures, the phenotype and metabolic profiling of these mutant strains remained consistent. One of the positive transformants was used for further analysis.

### 3.2. Analysis of Phenotypic and Secondary Metabolite Profile of ΔhdaA Mutant of A. terreus RA2905

**Figure 2:**
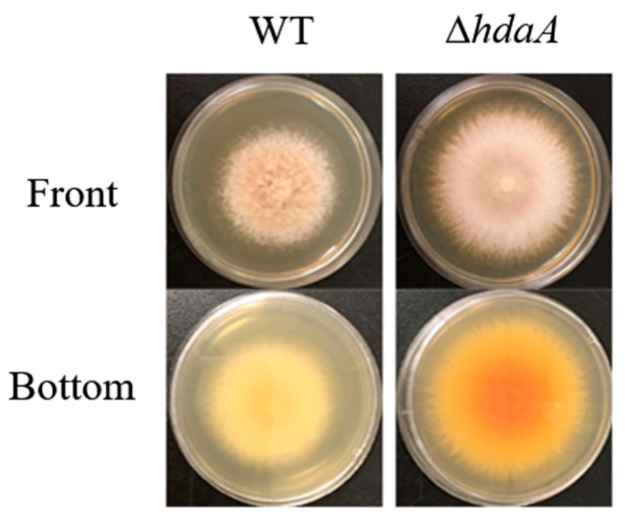
Growth of wild-type (WT) *A. terreus* RA2905 and the ∆*hdaA* strain on PDA plates.

**Figure 3:**
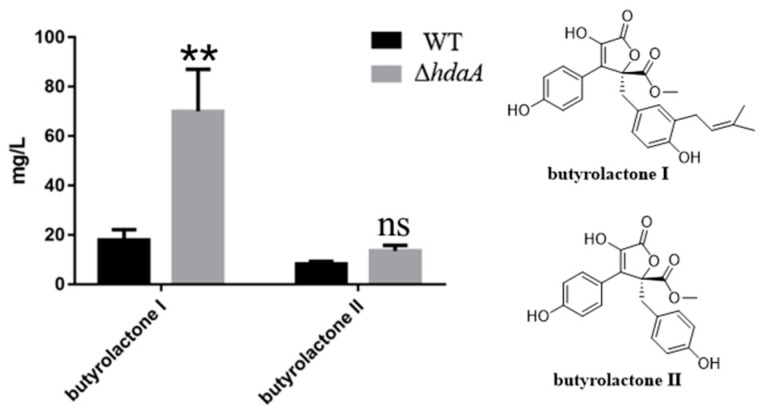
Analysis of the production levels of butyrolactones I and II in the wild-type (WT) strain and Δ*hdaA* strain. Titers of butyrolactones I and II were the means of three biological replicates. ns. means no significance, ** *p*  ≤  0.01.

**Figure 4:**
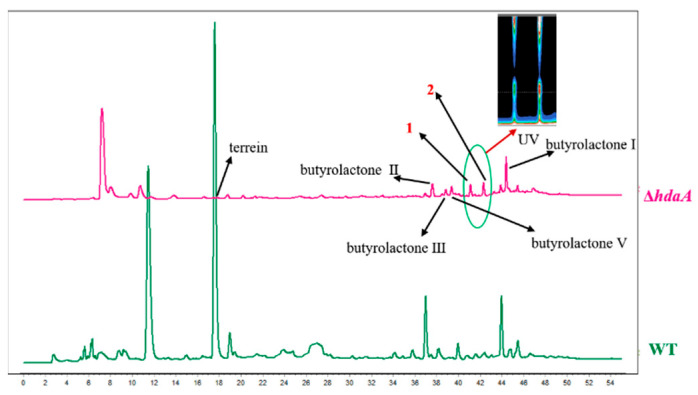
HPLC analysis of the secondary metabolite profiles of WT strain and ∆*hdaA* strain.

**Figure 5:**
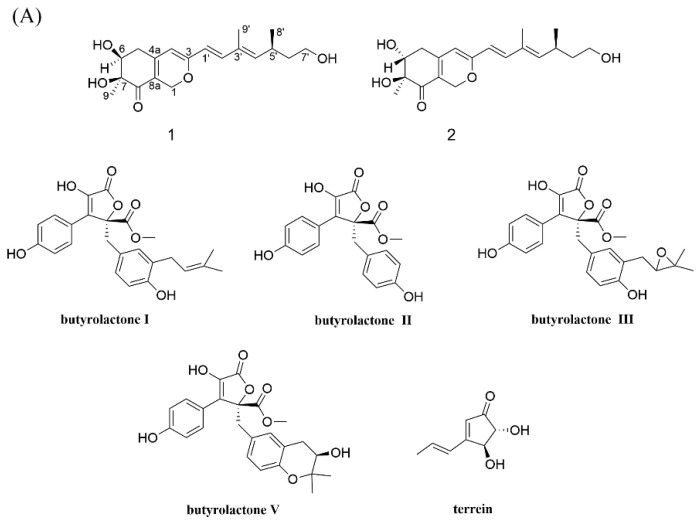

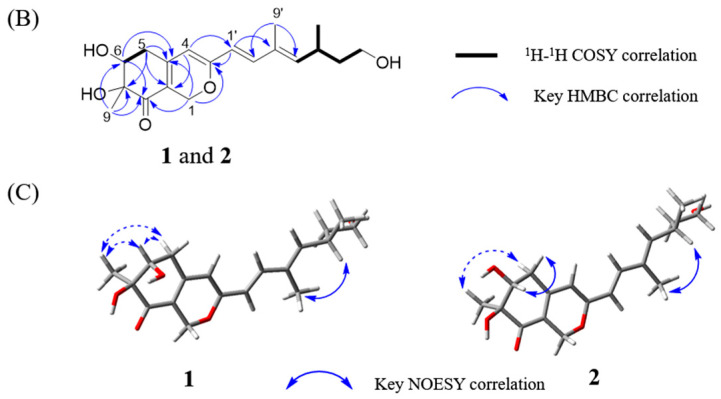
(**A**) The structures of compounds **1** and **2** and known compounds. (**B**) Key ^1^H-^1^H COSY and HMBC correlations of **1** and **2**. (**C**) Key NOESY correlations of **1** and **2**.

The Δ*hdaA* mutant strain displayed phenotypic differences compared to WT when cultured in solid potato dextrose media (PDA). The Δ*hdaA* mutant produced approximately 50% more conidia than WT, and a sienna pigment emerged in Δ*hdaA* mutant but not in WT (Figure 2). The HPLC analysis of EtOAc extracts of the Δ*hdaA* mutant and the WT cultured in potato broth showed that loss of HdaA greatly altered the secondary metabolic profile of *A. terreus* RA2905. The *hdaA* deletion mutant synthesized three folds more butyrolactone I than WT (Figure 3), indicating that *hdaA* has a negative regulatory effect on the biosynthesis of butyrolactone I in *A. terreus*. It should be mentioned that TLC analysis exhibited two new fluorescent yellow spots in the fungal extract of Δ*hdaA*, but not in WT. Correspondingly, the HPLC profiles confirmed that the Δ*hdaA* mutant produced new peaks at 41.0 and 42.4 min with distinctive UV maximum absorption wavelengths at 260 and 400 nm (Figure 4 and Figure S17), which are not observed in WT. Then, the Δ*hdaA* mutant was cultivated in a large scale to attempt the attainment of these newly produced compounds. From the EtOAc extract, five known compounds, including four butyrolactones (butyrolactones I−III, V) and one polyketide terrein (Figure 5), were separated by a combination column chromatography on silica gel and an ODS. Meanwhile, two new compounds (**1** and **2**, Figure 5) were isolated using an ODS column eluted with 60% MeOH−H_2_O under the guidance of TLC with two fluorescent yellow spots, which were exactly the corresponding differential absorption peaks determined by HPLC analysis. Herein, compounds **1** and **2** were given the names asperterilone A and asperterilone B, respectively.

### 3.3. Structure Elucidation

Asperterilone A (**1**) was isolated as a yellow oil. Its molecular formula of C_19_H_26_O_5_ was deduced from its HRESIMS [*m/z* 335.1845 [M+H]^+^ (calcd 335.1853)] (Figure S8) and 1D NMR data analysis (Figures S1−S3), suggesting seven degrees of unsaturation. The ^1^H NMR spectroscopic data (Table 2) revealed three methyl groups, four olefinic protons, four methylene groups (one linked to unsaturated carbon [*δ*_H_ 2.63 (dd, *J* = 18.6, 3.9 Hz, H_e_-5); *δ*_H_ 2.83 (ddd, *J* = 18.6, 3.6, 1.3 Hz, H_a_-5)]; two oxygenated [*δ*_H_ 3.48 (dt, *J* = 10.6, 7.0 Hz, H-7′a); *δ*_H_ 3.53 (dt, *J* = 10.6, 7.0 Hz, H-7′b); *δ*_H_ 4.80 (d, *J* = 12.6, H-1a); *δ*_H_ 4.94 (d, *J* = 12.6 Hz, H-1b)], and two methines (one oxygenated [*δ*_H_ 3.99 (t, *J* = 3.9 Hz, H-6)]. The ^13^C NMR and HSQC data revealed the presence of 19 carbons, including three methyls, four methylenes (including two *O*-methylene), two methines (including one *O*-methine), one oxygenated quaternary carbon, eight olefinic carbons accounting for four olefin groups, and a ketone carbon (*δ*_C_ 196.5). These data accounted for five of the seven unsaturation degrees, indicating **1** as a dicyclic compound. The NMR signals indicated that **1** was equipped with a featured skeleton as azaphilone characterized with oxygenated unsaturated carbon C-3, oxygenated nonprotonated carbon C-7, quaternary carbon C-4a and C-8a, and ketone carbon C-8.

In ^1^H-^1^H COSY spectrum of **1** (Figure S4), the correlations among H-4′/H-5′/H-6′/H-7′/H-8′ contributed to the partial side chain C-4′-C-8′ moiety. The HMBC correlations (Figure S6) from H-9′ to C-2′ and C-4′, and from H-1′ to C-3 and C-3′ indicated the side chain containing a diene structure. The HMBC connections from H-5 to C-4a, C-7, and C-8a, and from H-6 to C-4a and C-8 revealed the presence of a cyclohexenone ring. Furthermore, the HMBC correlations from H-9 to C-6, C-7, and C-8 indicated that the methyl group was anchored at C-7. The HMBC cross peaks from H-4 to C-1′ and C-8a, and from H-1 to C-3, C-4a, and C-8 established the oxygenated six-membered ring with the side chain attached at C-3, which fused with the former cyclohexenone ring. Therefore, the planar structure of compound **1** was established as shown in Figure 5.

Asperterilone B (**2**) was also isolated as a yellow oil. The molecular formula of **2** was determined as C_19_H_26_O_5_ on the basis of its positive HRESIMS (Figure S16) at *m/z* 335.1847 [M+H]^+^ (calcd 335.1853), the same as that of compound **1**. The ^1^H NMR and ^13^C NMR spectroscopic data of **2** (Figures S9−S11) were similar to those of **1**. Careful analysis of the NMR data revealed the apparent differences of chemical shifts of H-5 (*δ*_H_ 2.48 and 2.70 in **2** vs. *δ*_H_ 2.63 and 2.83 in **1**), and H-9 (*δ*_H_ 1.24 in **2** vs. *δ*_H_ 1.31 in **1**); C-6 (*δ*_C_ 72.2 in **2** vs. *δ*_C_ 73.7 in **1**), C-7 (*δ*_C_ 76.8 in **2** vs. *δ*_C_ 75.5 in **1**), especially C-9 (*δ*_C_ 16.8 in **2** vs. *δ*_C_ 21.6 in **1**). It was suggested that the difference took place at the cyclohexenone ring which was probably caused by the configuration change at C-6 or C-7. The relative configurations of compounds **1** and **2** were deduced by analysis of their ^1^H NMR *J*-values and NOESY data. As for compound **1**, the small coupling constants (*J*_5a,6_ = 3.6 Hz, *J*_5e,6_ = 3.9 Hz) observed between H_2_-5 and H-6 indicated that H-6 occupied an equatorial position. Based on the ^13^C NMR-based empirical rules [28,29], the down field-shift of C-9 (*δ*_C_ 21.6) demonstrated that CH_3_-9 was in an anti-*γ*-gauche effect relationship with OH-6, indicating that CH_3_-9 was in an axial position. The NOESY correlations (Figure S7) between H_3_-9/H_a_-5/H-6 confirmed that the relative configurations of **1** were the same as the mentioned rules. In compound **2**, the large coupling constant between H-6/H_a_-5 (*J* = 9.8 Hz) revealed that H-6 occupied an axial position. In accordance with the ^13^C NMR-based empirical rules [28], CH_3_-9 was in a *γ*-gauche effect relationship with OH-6, which caused C-9 to shift to a higher field of *δ*_C_ 16.8. These findings confirmed that CH_3_-9 was in an axial orientation. In the NOESY spectrum (Figure S15), NOE correlations from H_3_-9 to H_a_-5 and from H-6 to H_e_-5 revealed that H-6 and H_3_-9 were on the opposite side, which was consistent with the mentioned rules. The configuration of the double bond C-1′−C-2′ was determined as *E* based on the large coupling constant between H-1′ and H-2′ (*J* = 15.5 Hz). In the NOESY spectra, the correlations observed between H-5′ and H_3_-9′ suggested the double bond between C-3′ and C-4′ had the *E*-configuration.

**Scheme 1:**
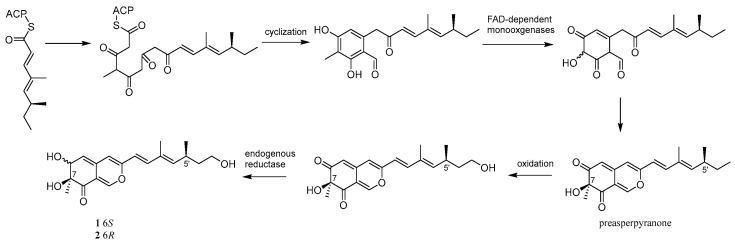
Possible pathway for the biosynthesis of compounds **1** and **2**.

The absolute configurations of the compounds were determined by comparing their CD spectra with those of similar compounds reported in the literature and the presumed biosynthetic pathway. The CD spectra of **1** and **2** (Figure S18) showed consistent positive [+5.32 (**1**), +3.22 (**2**) at 338 nm] and negative [−3.80 (**1**), −0.30 (**2**) at 256 nm] Cotton effects which contrast with those of the nigirpexin C (negative at 338 nm and positive at 247 nm), a pulvilloric acid-type azaphilone produced by *Nigrospora oryzae* co-cultured with *Irpex lacteus* [30], indicating that compounds **1** and **2** possessed the same *S*-configuration at C-7. As mentioned above, the relative configurations of the cyclohexenone rings of compounds **1** and **2** have been unambiguously determined, thus, the absolute configurations of **1** and **2** were determined to be 6*S*,7*S* and 6*R*,7*S*, respectively. Biosynthetically, these two compounds originated from the preasperpyranone biosynthetic pathway, which was derived from *A. terreus* MEFC01 [5]. The plausible biosynthetic pathway was suggested as shown in Scheme 1, revealing that compounds **1** and **2** were yielded by the oxidation and reduction in preasperpyranone. Therefore, the stereochemistry of C-5’ was assigned as 5’*S* in **1** and **2** considering the shared biosynthetic pathway. Eventually, the absolute configurations of **1** and **2** were assigned as 6*S*,7*S*,5’*S* and 6*R*,7*S*,5’*S*, respectively. Therefore, compounds **1** and **2** are a pair of C-6 isomers.

### 3.4. Spectroscopic and Spectrometric Data

Asperterilone A (**1**): Yellow oil; [α]^20^_D_ +29.8 (*c* 1.0, MeOH); UV (MeOH) *λ*_max_ (log *ε*) 199 (2.17), 230 (1.88), 259 (1.42), 396 (0.90) nm; ECD (0.5 mM, MeOH), *λ*_max_ (Δ*ε*) 256 (−3.8), 338 (+5.32) nm; IR (KBr) *ν*_max_ 3387, 2942, 1708, 1385, 1268, 1029 cm^−1^; ^1^H and ^13^C NMR data, see Table 2; HRESIMS *m/z* 335.1845 [M+H]^+^ (calcd for C_19_H_27_O_5_, 335.1853).

Asperterilone B (**2**): Yellow oil; [α]^20^_D_ −15.6 (*c* 1.0, MeOH); UV (MeOH) *λ*_max_ (log *ε*) 199 (1.44), 230 (0.79), 259 (0.57), 396 (0.41) nm; ECD (0.4 mM, MeOH), *λ*_max_ (Δ*ε*) 256 (−0.3), 338 (+3.22) nm; IR (KBr) *ν*_max_ 3435, 2924, 1627, 1384, 1166, 1053 cm^−1^; ^1^H and ^13^C NMR data, see Table 2; HRESIMS *m/z* 335.1847 [M+H]^+^ (calcd for C_19_H_27_O_5_, 335.1853).

### 3.5. Bioassays of Compounds

#### 3.5.1. Anti-Fungal Activity Assay

In this research, anti-*Candida* activities of **1** and **2** were tested against three species of pathogenic *Candida* species, including *C. albicans* ATCC 24433, *C. parapsilosis* ATCC 22019, and *C. tropicalis* ATCC 20962. The results showed that **1** and **2** exhibited moderate activities against *C. parapsilosis* with the MIC values of 18.0 and 23.9 μM, respectively (MIC value of amphotericin, 2.2 μM). Additionally, both compounds displayed weak anti-*Candida* activities against *C. albicans* with the same MIC values of 47.9 μM. However, **1** and **2** showed no activity toward *C. tropicalis*.

#### 3.5.2. Cytotoxicity Assay

The primary screening of cytotoxic activity was carried out using the human breast cancer cell line MDA-MB-231. The results revealed that compound **1** with 6*S*-configuration exhibited significant cytotoxic activity with 81.1% inhibition at 10 μM, while compound **2** with 6*R*-configuration showed no activity under the same concentration. It is worth noting that this pair of epimers exhibited differential cytotoxic activity, suggesting that the configuration at C-6 may play a critical role in cytotoxicity.

## 4. Discussion

During the last decade, HdaA as a major class 2 HDAC has been extensively studied in many fungi. For example, in *A. nidulans*, deletion of the *hdaA* gene resulted in the loss of the main activity peak and in a dramatic reduction in total HDAC activity [31]. Moreover, Δ*hdaA* strains displayed a remarkable reduction in growth under different conditions of oxidative stress. The loss of *hdaA* in *A. niger* activated pigment synthesis in liquid CD medium cultivation [8]. In *A. fumigatus*, the *hdaA* was demonstrated to closely correlate with the regulation of secondary metabolite production [7]. Therefore, HdaA was assumed to be a main contributor to HDAC activity and a global regulator of secondary metabolism in *Aspergillus*. However, *hdaA* has never been identified in *A. terreus*. Of these eight histone deacetylase genes, *hdaA* showed the highest expression level under PDB media (a common condition for inducing secondary metabolite production). Accordingly, we explored whether HdaA plays a role in the regulation of secondary metabolites biosynthesis. Indeed, as the above results showed, the deletion of *hdaA* not only improved butyrolactones production, but also activated a new gene cluster, resulting in the discovery of new azaphilones, which further expand the pool of azaphilones from nature. Azaphilones as an important class of natural products exhibit a wide range of excellent pharmacological activities, such as antimicrobial, antiviral, cytotoxic, anticancer, and anti-inflammatory activities [31,32,33,34]. It should be highlighted that the azaphilones exploited by genomic mining, asperterilones A and B (**1** and **2**), exhibited selective antifungal activity against *C. parapsilosis* and *C. albicans*. Unexpectedly, these two compounds have no activity target of *C. tropicalis*. The detailed mechanism for this selectivity deserved further investigation. *A. terreus* has been proven to have great potential in producing azaphilone compounds, such as azasperpyranone A, an azaphilone possessing a 6/6/6/6 tetracyclic ring system synthesized by two separate clusters collaboratively [5]. However, azaphilone compounds have not been found in the wild-type strain of *A. terreus* RA2905 cultured in many types of media in our previous analysis.

In the past few years, chemical epigenetic modification has been widely used in the discovery of secondary metabolites of marine-derived fungi. However, in many filamentous fungi, this strategy was not always effective, otherwise the changes in secondary metabolism induced by chemical epigenetic modification had poor reproducibility. The reason could be due to the epigenetic agents used which may not be specific to fungal HDACs or may even be degraded by the fungi [35]. As a result, in our study, we provided an alternative to awaken the silent secondary metabolites. The most critical HDAC was identified first, then genetically stable mutants were acquired through gene knockout or mutation, followed by targeted isolation of the newly emerging chemical molecules. The strategy greatly improved the efficiency of discovering new compounds.

In conclusion, this work provided the first insight into the epigenetic regulation mediated by HdaA in the biosynthesis of secondary metabolites in *A. terreus*. Additionally, two new azaphilone compounds with anti-*Candida* activity were obtained from the *hdaA* deletion mutant.

## Figures and Tables

**Table 1:** The expression level of HDAC genes in *A. terreus* when cultured in PDB liquid media.

Gene	Amino Acids	Homolog	FPKM
*gene0245*	490 aa	Hst4	16
*gene3853*	1068 aa	Sir2	13
*gene4092*	500 aa	Sds3	35
*gene8076*	760 aa	RPD3	27
*gene9509*	484 aa	HosA	20
*gene9554*	675 aa	RPD3	0
*gene9818*	760 aa	Hda1	500
*gene10107*	1245 aa	HosB	2

FPKM: Fragments per kilobase of transcript per million fragments mapped.

**Table 2:** ^1^H (500 MHz) and ^13^C (125 MHz) chemical shifts for identified compounds **1** and **2** in methanol-*d*_4_.

Position	1			2	
	*δ*_C_, Type	*δ*_H_, Mult. (*J* in Hz)		*δ*_C_, Type	*δ*_H_, Mult. (*J* in Hz)
1	63.4, CH_2_	4.80, d (12.6) 4.94, d (12.6)		63.5, CH_2_	4.82, d (12.6) 4.92, d (12.6)
3	161.0, C			161.1, C	
4	105.1, CH	5.53, s		104.7, CH	5.56, s
4a	148.6, C			150.0, C	
5	33.4, CH_2_	H_e_ 2.63, dd (18.6, 3.9) H_a_ 2.83, ddd (18.6, 3.6, 1.3)		34.1, CH_2_	H_a_ 2.48, ddd (17.8, 9.8, 1.7) H_e_ 2.70, dd (17.8, 5.4)
6	73.7, CH	3.99, t (3.9)		72.2, CH	3.93, dd (9.8, 5.4)
7	75.5, C			76.8, C	
8	196.5, C			197.3, C	
8a	113.6, C			114.1, C	
9	21.6, CH_3_	1.31, s		16.8, CH_3_	1.24, s
1′	118.6, CH	6.07, d (15.5)		118.5, CH	6.07, d (15.5)
2′	139.4, CH	6.88, d (15.5)		140.0, CH	6.92, d (15.5)
3′	132.6, C			132.6, C	
4′	144.4, CH	5.56, d (9.8)		144.6, CH	5.58, d (9.8)
5′	29.4, CH	2.76, m		29.4, CH	2.79, m
6′	39.6, CH_2_	1.52, m 1.61, m		39.6, CH_2_	1.52, m 1.62, m
7′	59.6, CH_2_	3.48, dt (10.6, 7.0) 3.53, dt (10.6, 7.0)		59.6, CH_2_	3.48, dt (10.6, 7.0) 3.55, dt (10.6, 7.0)
8′	19.5, CH_3_	1.03, d (6.7)		19.5, CH_3_	1.02, d (6.7)
9′	11.1, CH_3_	1.82, s		11.1, CH_3_	1.83, s

## Data Availability

Not applicable.

## Supplementary Materials

The following supporting information can be downloaded at: https://www.mdpi.com/article/10.3390/jof8101024/s1. Figures S1–S16: ^1^H NMR, ^13^C NMR, HSQC, ^1^H-^1^H COSY, HMBC, and HRESIMS spectra of compounds **1** and **2**. Figure S17: HPLC detection on ethyl acetate (EtOAc) extracts of ∆*hdaA* strain (A) and WT strain (B). Figure S18: CD curves of compounds **1** and **2**. Figure S19: The PCR conditions for construction of the mutant.
