# Supplementary material for: Induction of Secondary Metabolite Biosynthesis by Deleting the Histone Deacetylase HdaA in the Marine-Derived Fungus Aspergillus terreus RA2905
Source: J Fungi (Basel). 2022 Sep 28;8(10):1024. doi: 10.3390/jof8101024 (PMC9604699; doi:10.3390/jof8101024)
Supplement: Supplementary file 1 [file jof-08-01024-s001.zip › jof-1914799-supplementary.pdf]

List of Supporting Information

Figure S1. ^{1}H NMR spectrum of compound **1** in methanol-d_4 (500 MHz).
Figure S2. Partial ^{1}H NMR spectrum of compound **1** in methanol-d_4 (500 MHz).
Figure S3. ^{13}C NMR spectrum of compound **1** in methanol-d_4 (125 MHz).
Figure S4. ^{1}H-^{1}H COSY spectrum of compound **1** in methanol-d_4.
Figure S5. HSQC spectrum of compound **1** in methanol-d_4.
Figure S6. HMBC spectrum of compound **1** in methanol-d_4.
Figure S7. NOESY spectrum of compound **1** in methanol-d_4.
Figure S8. HRESIMS spectrum (positive ion mode) of compound **1**.
Figure S9. ^{1}H NMR spectrum of compound **2** in methanol-d_4 (500 MHz).
Figure S10. Partial ^{1}H NMR spectrum of compound **2** in methanol-d_4 (500 MHz).
Figure S11. ^{13}C NMR spectrum of compound **2** in methanol-d_4 (125 MHz).
Figure S12. ^{1}H-^{1}H COSY spectrum of compound **2** in methanol-d_4.
Figure S13. HSQC spectrum of compound **2** in methanol-d_4.
Figure S14. HMBC spectrum of compound **2** in methanol-d_4.
Figure S15. NOESY spectrum of compound **2** in methanol-d_4.
Figure S16. HRESIMS spectrum (positive ion mode) of compound **2**.
Figure S17. HPLC detection on ethyl acetate (EtOAc) extracts of Δ*hdaA* strain (A) and WT strain (B).
Figure S18. CD curves of compounds **1** and **2**.
Figure S19. The PCR conditions for construction of mutant.

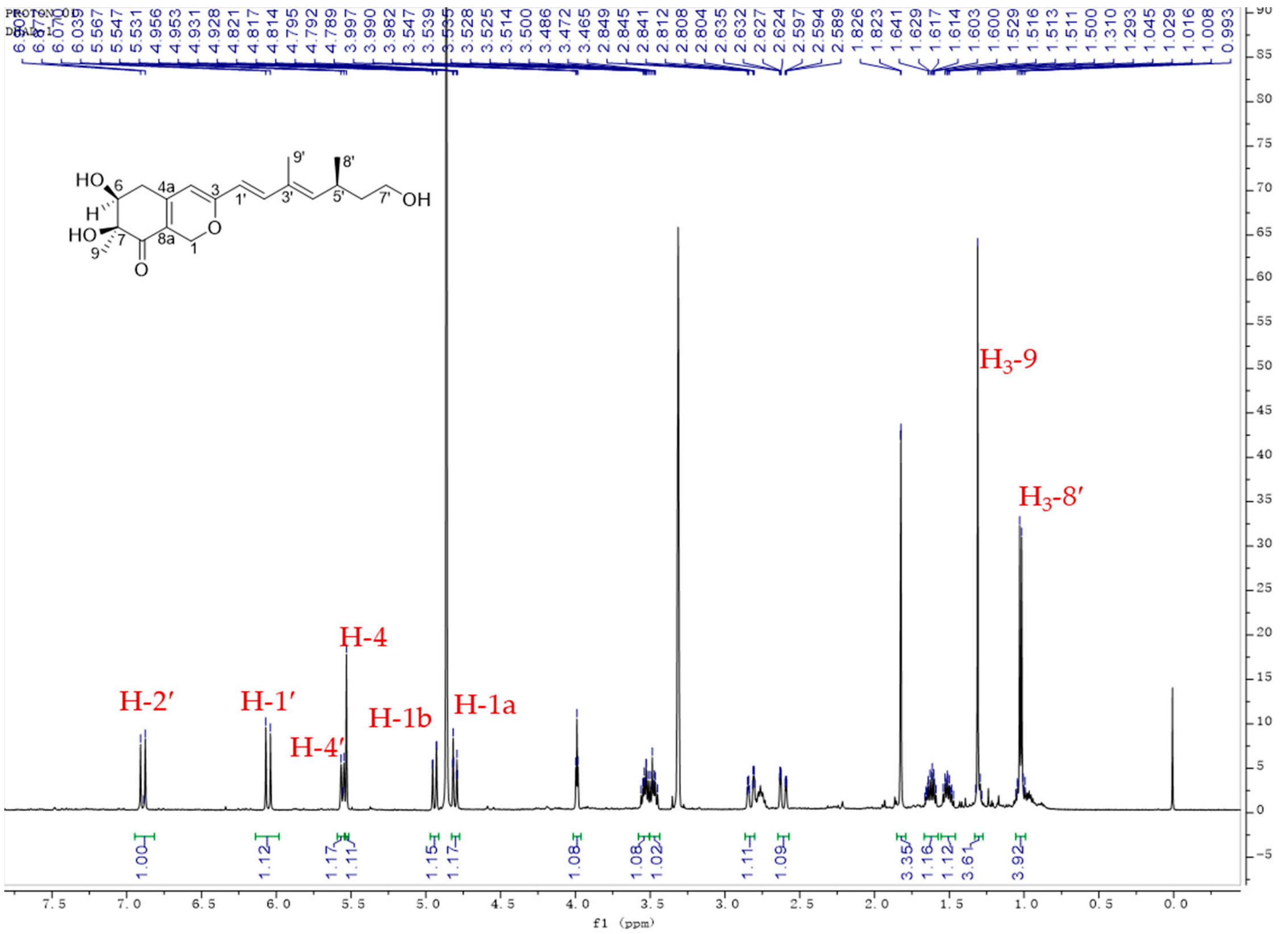

Figure S1. ^{1}H NMR spectrum of compound **1** in methanol-d_4 (500 MHz).

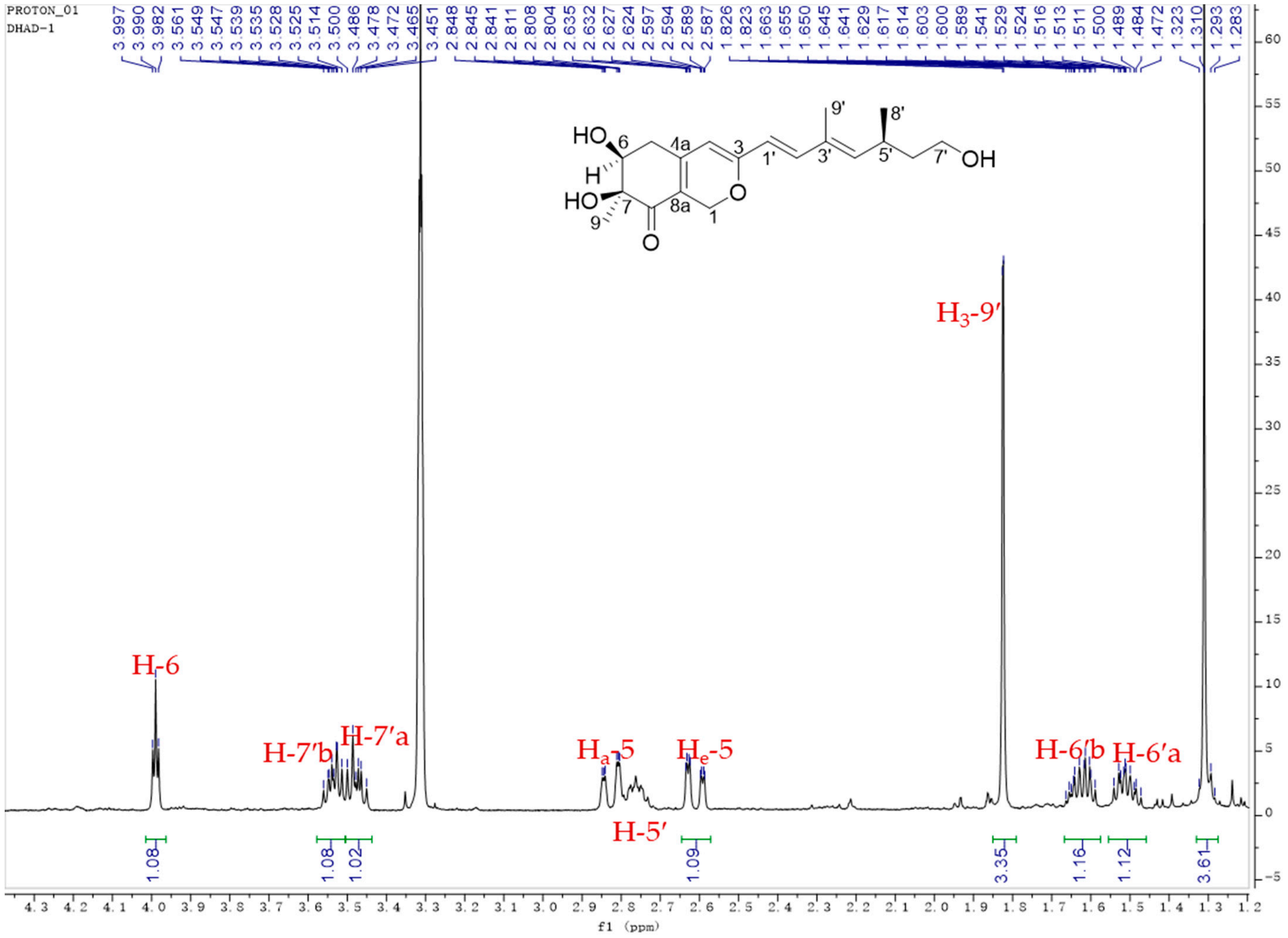

Figure S2. Partial ^{1}H NMR spectrum of compound **1** in methanol-d_4 (500 MHz).

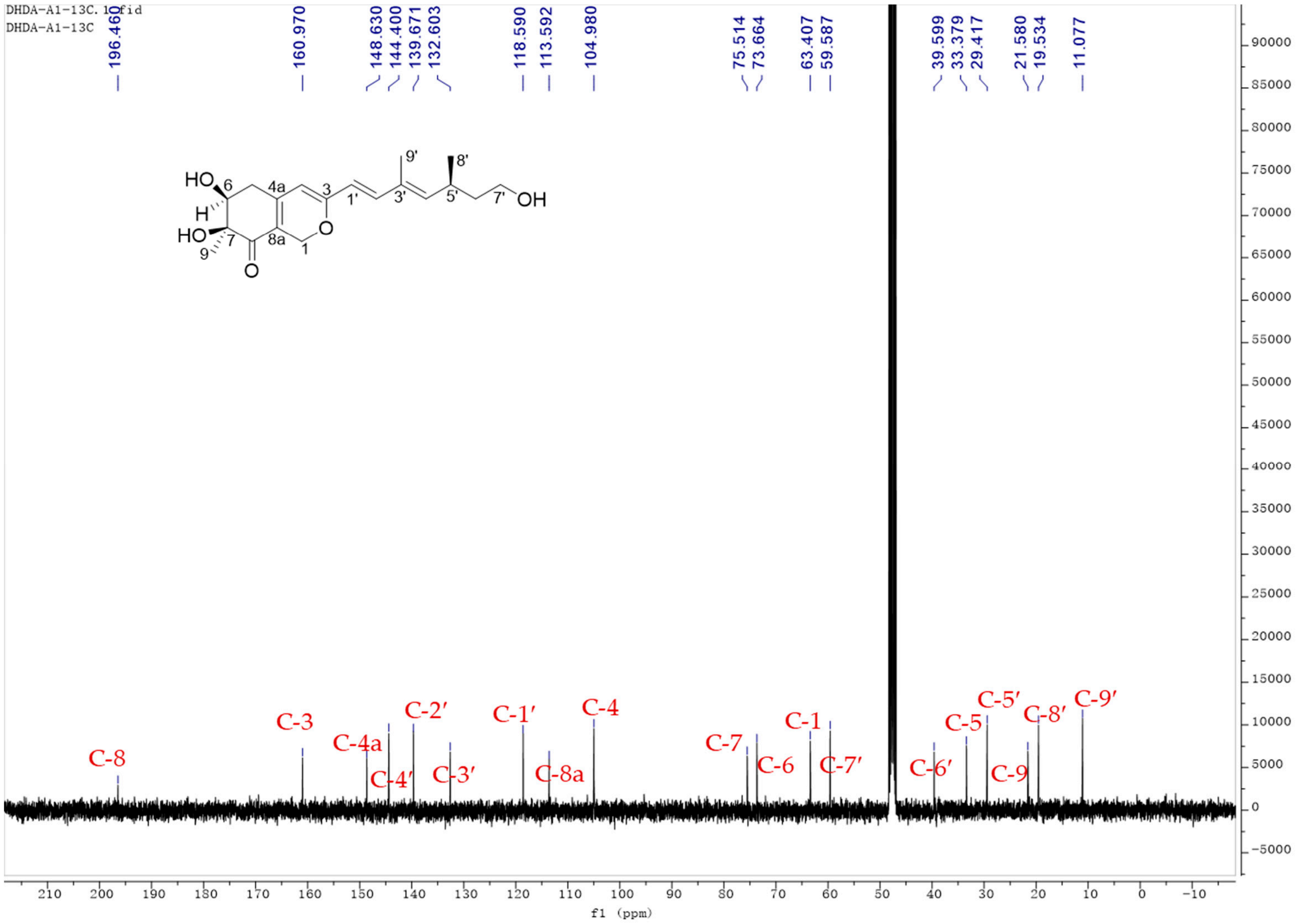

Figure S3. ^{13}C NMR spectrum of compound **1** in methanol-d_4 (125 MHz).

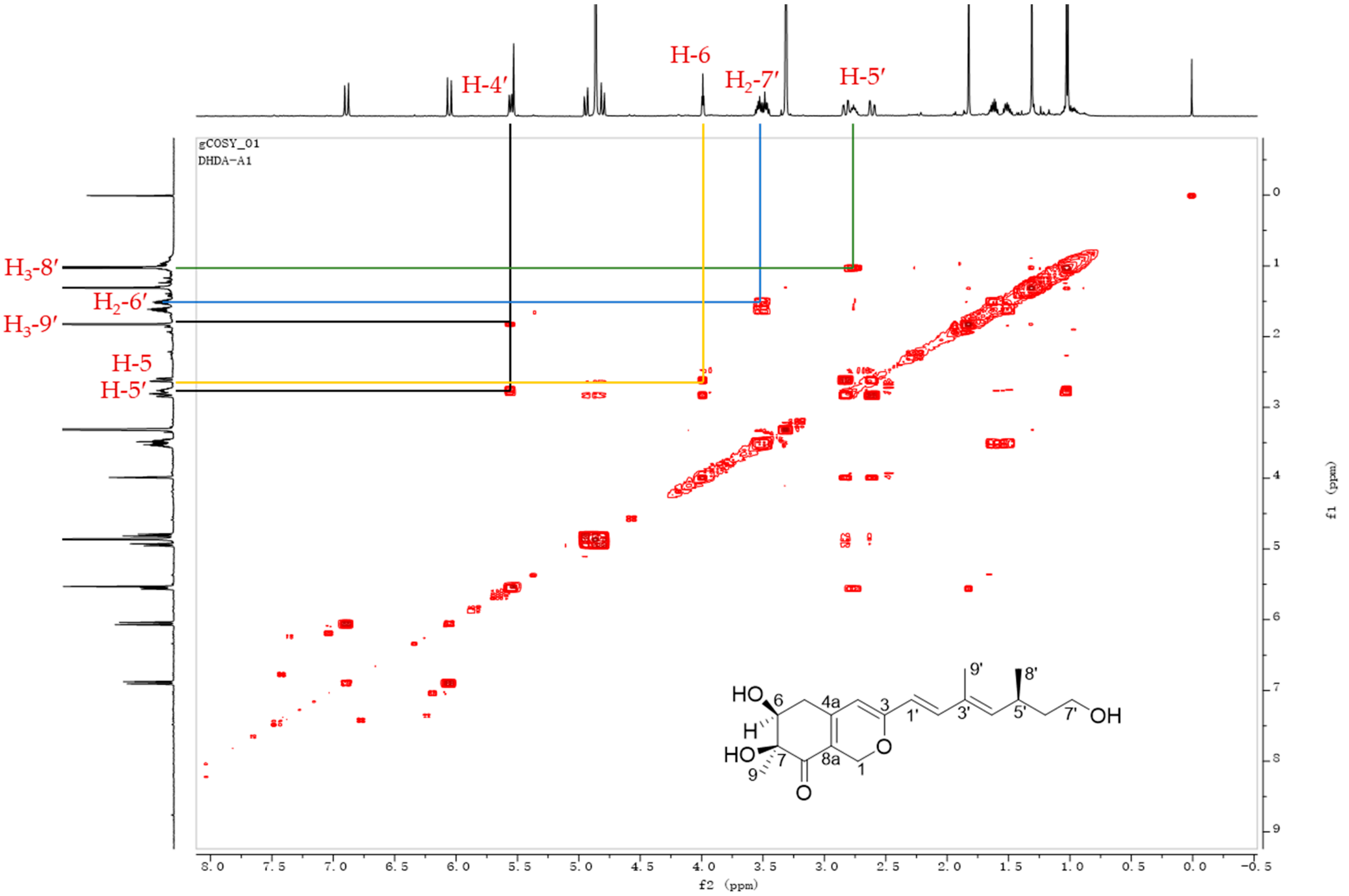

Figure S4. ^{1}H-^{1}H COSY spectrum of compound **1** in methanol-d_4.

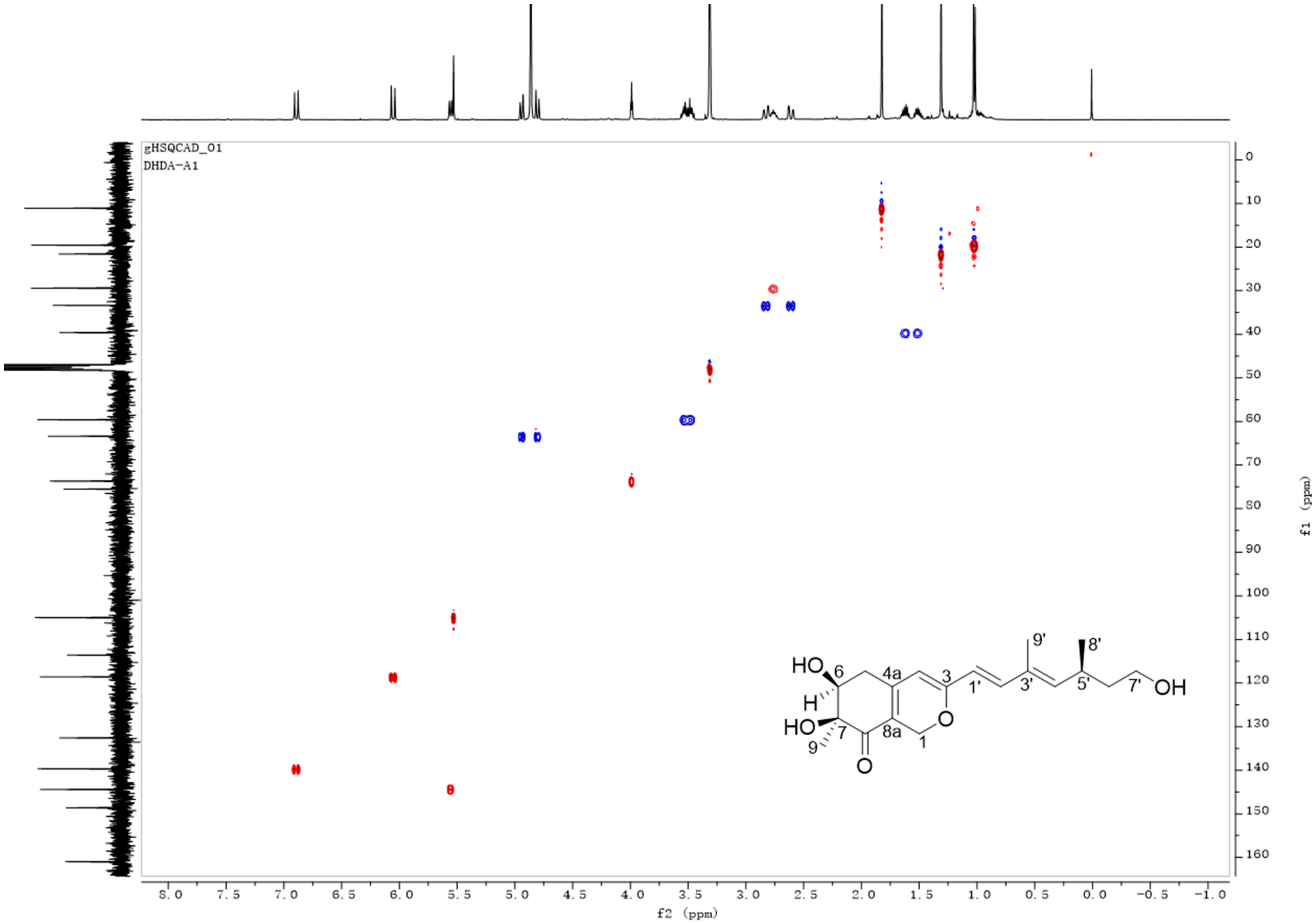

Figure S5. HSQC spectrum of compound **1** in methanol-d_4.

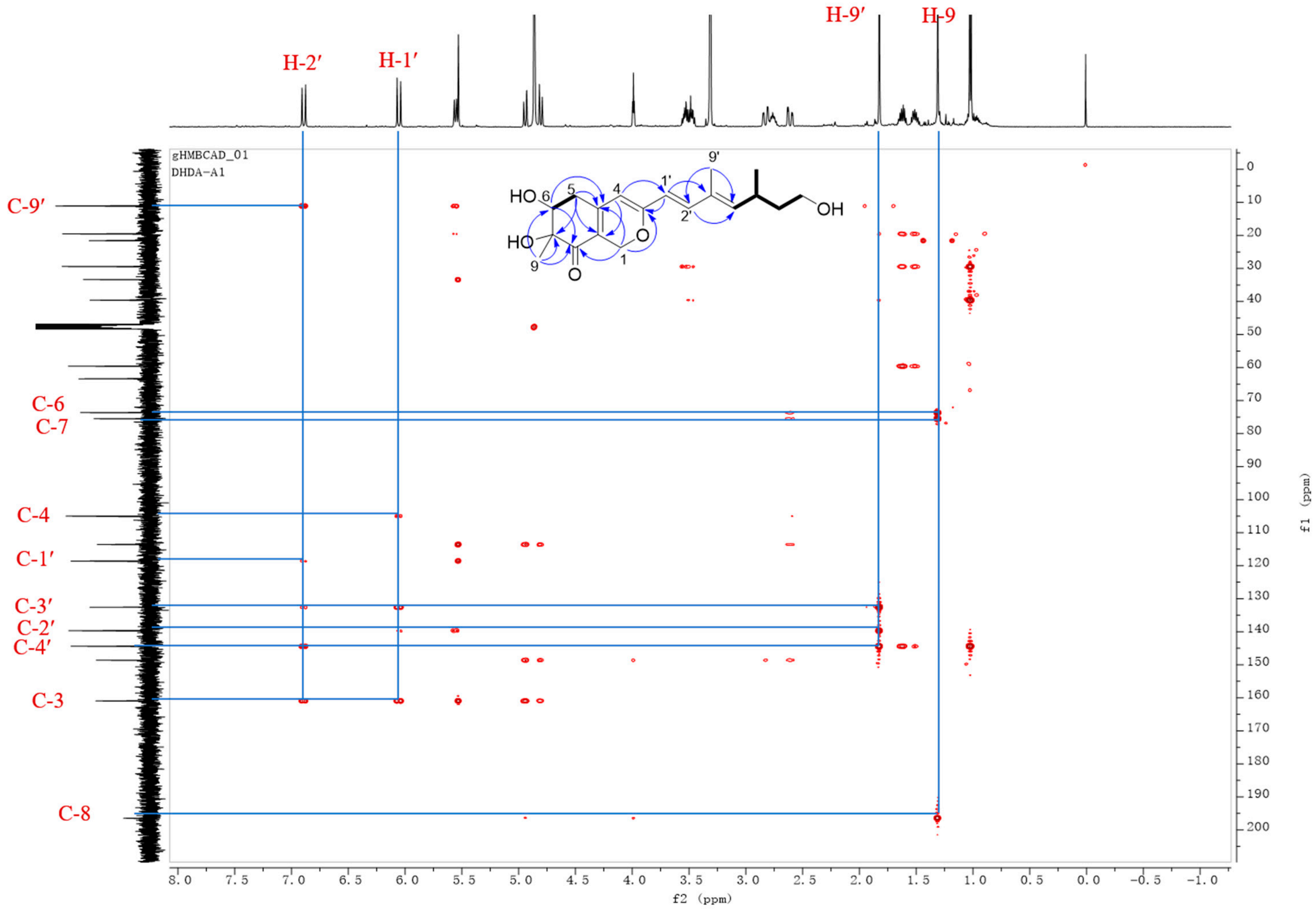

Figure S6. HMBC spectrum of compound **1** in methanol-d_4.

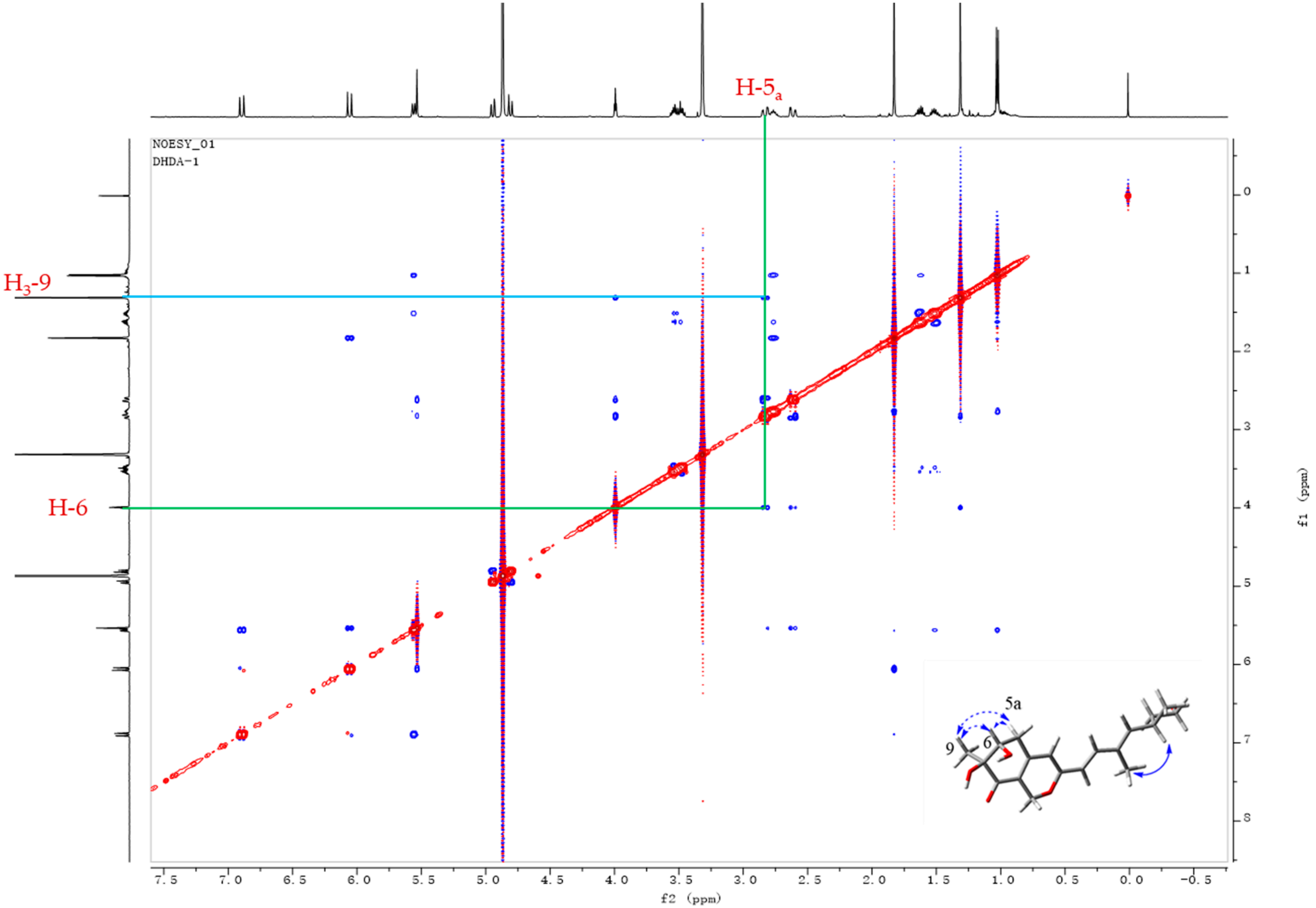

Figure S7. NOESY spectrum of compound **1** in methanol-d_4.

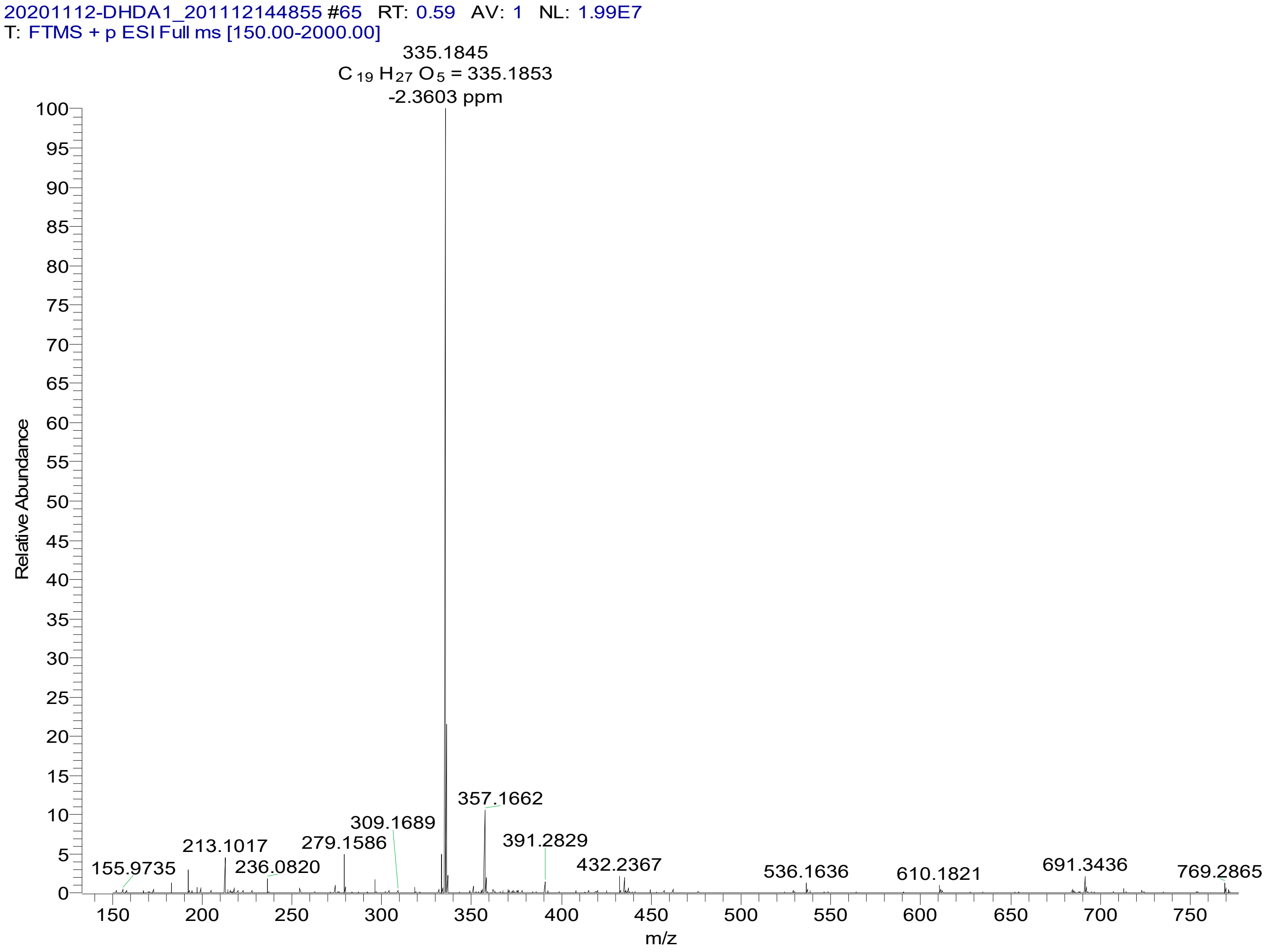

Figure S8. HRESIMS spectrum (positive ion mode) of compound **1**.

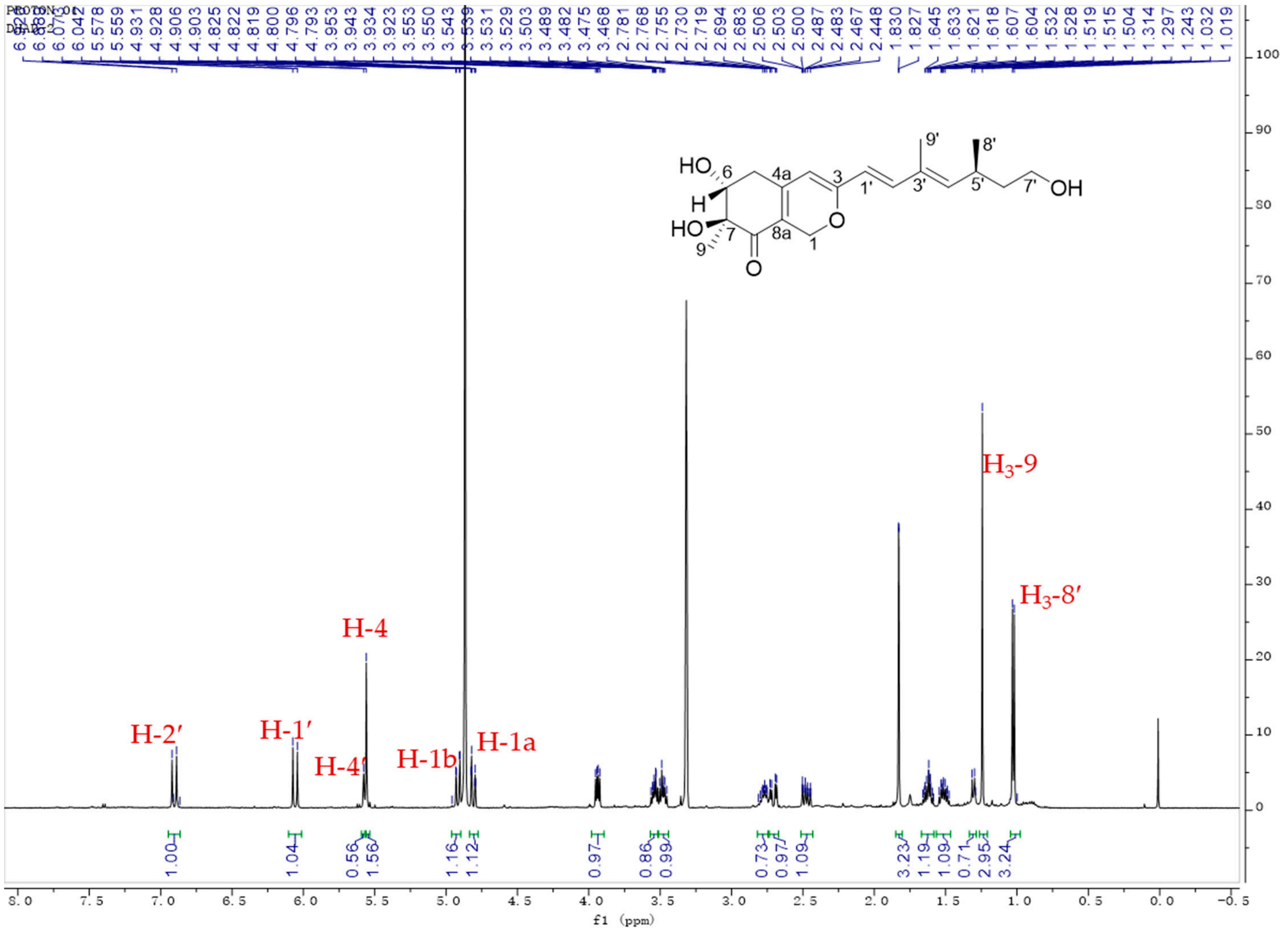

Figure S9. ^{1}H NMR spectrum of compound **2** in methanol-d_4 (500 MHz).

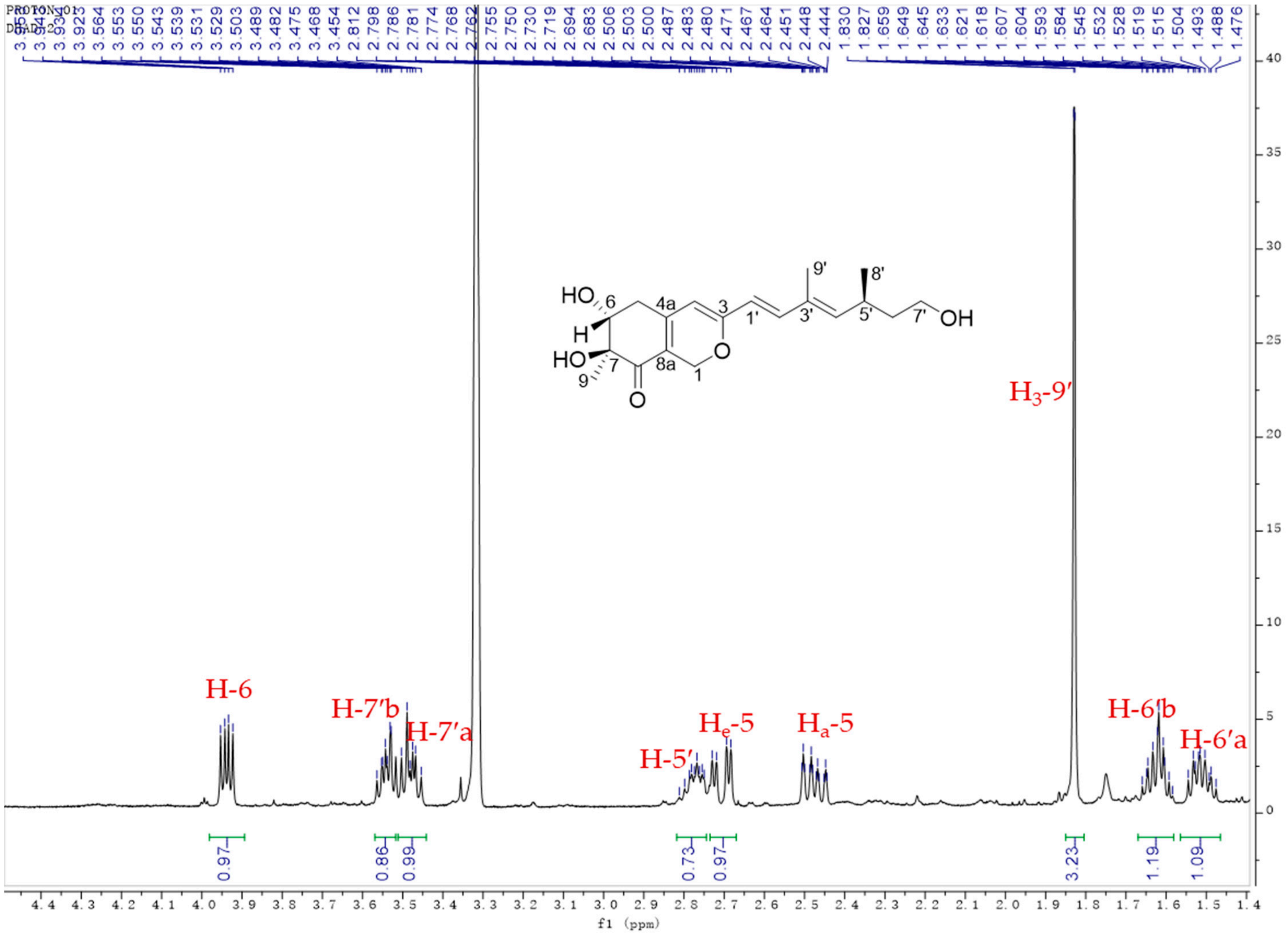

Figure S10. Partial ^{1}H NMR spectrum of compound **2** in methanol-d_4 (500 MHz).

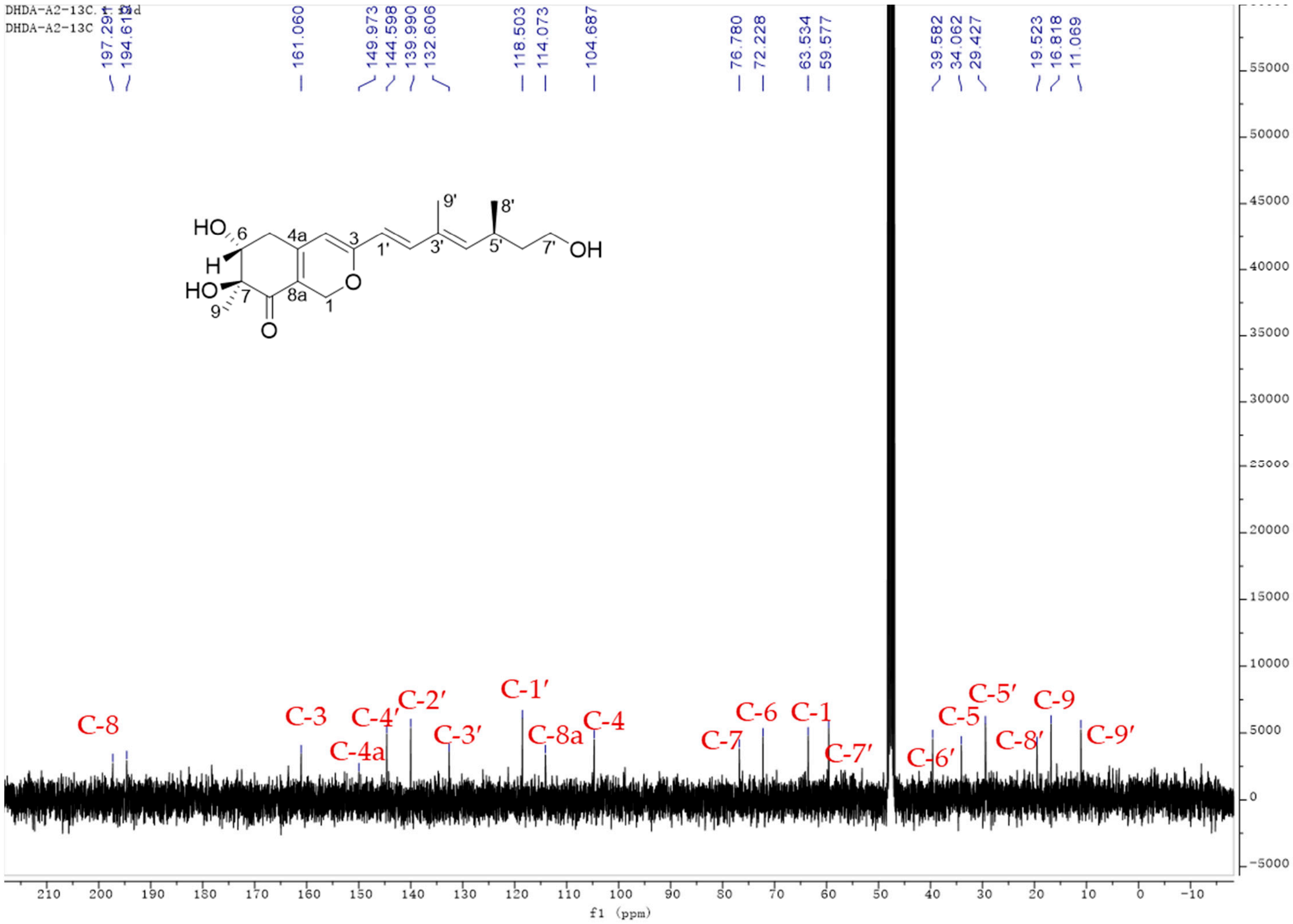

Figure S11. ^{13}C NMR spectrum of compound **2** in methanol-d_4 (125 MHz).

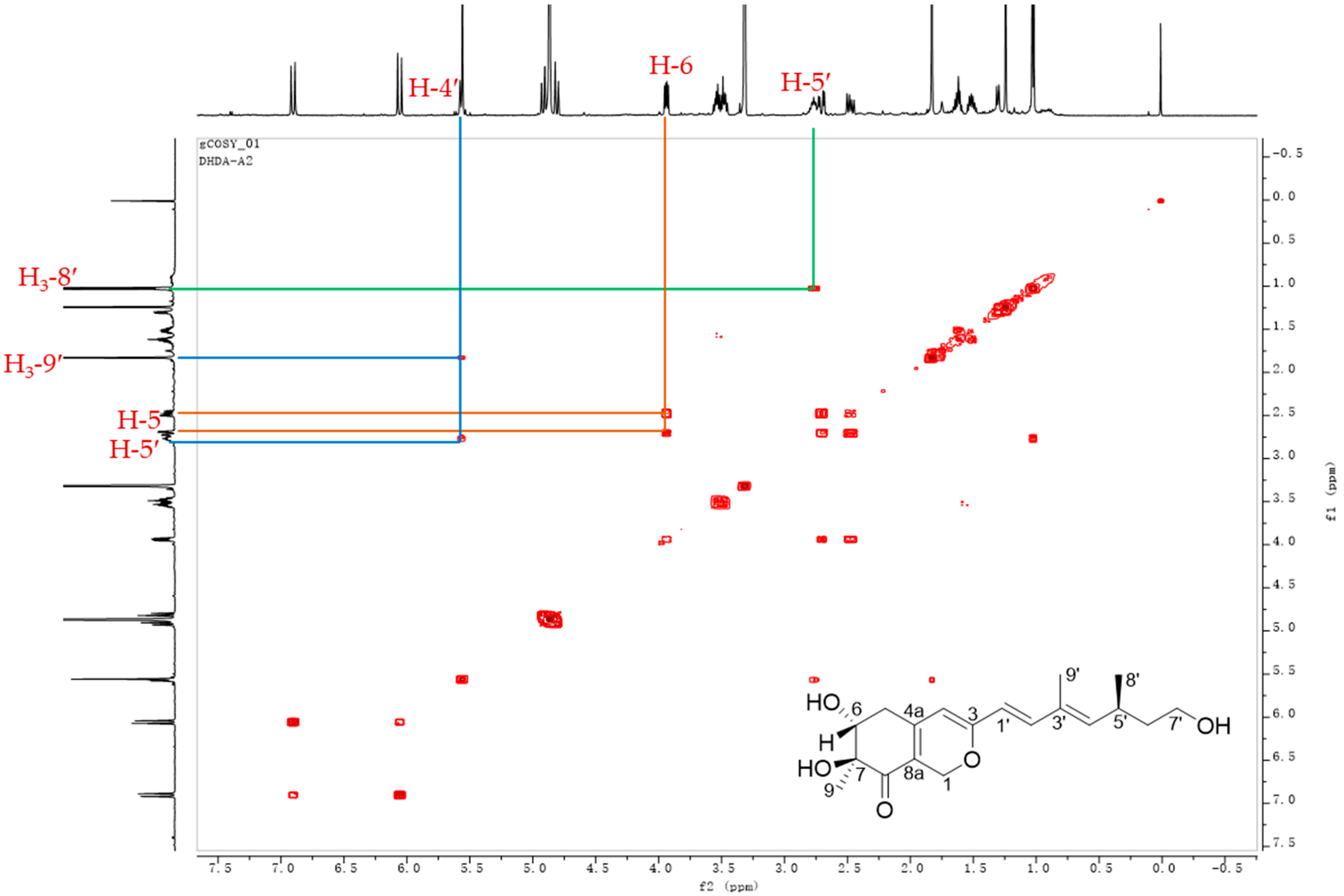

Figure S12. ^{1}H-^{1}H COSY spectrum of compound **2** in methanol-*d*$_4$.

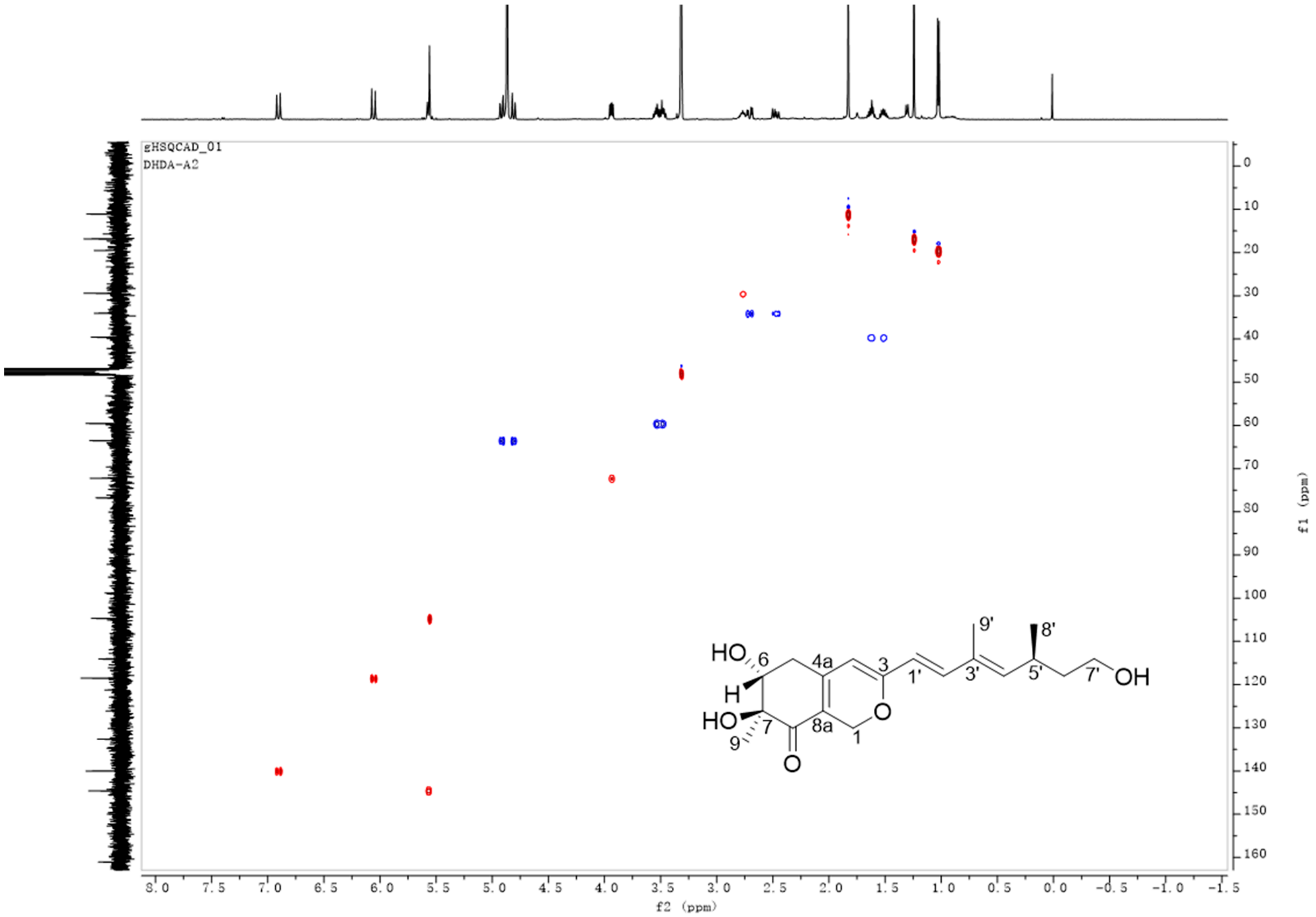

Figure S13. HSQC spectrum of compound **2** in methanol-d_4.

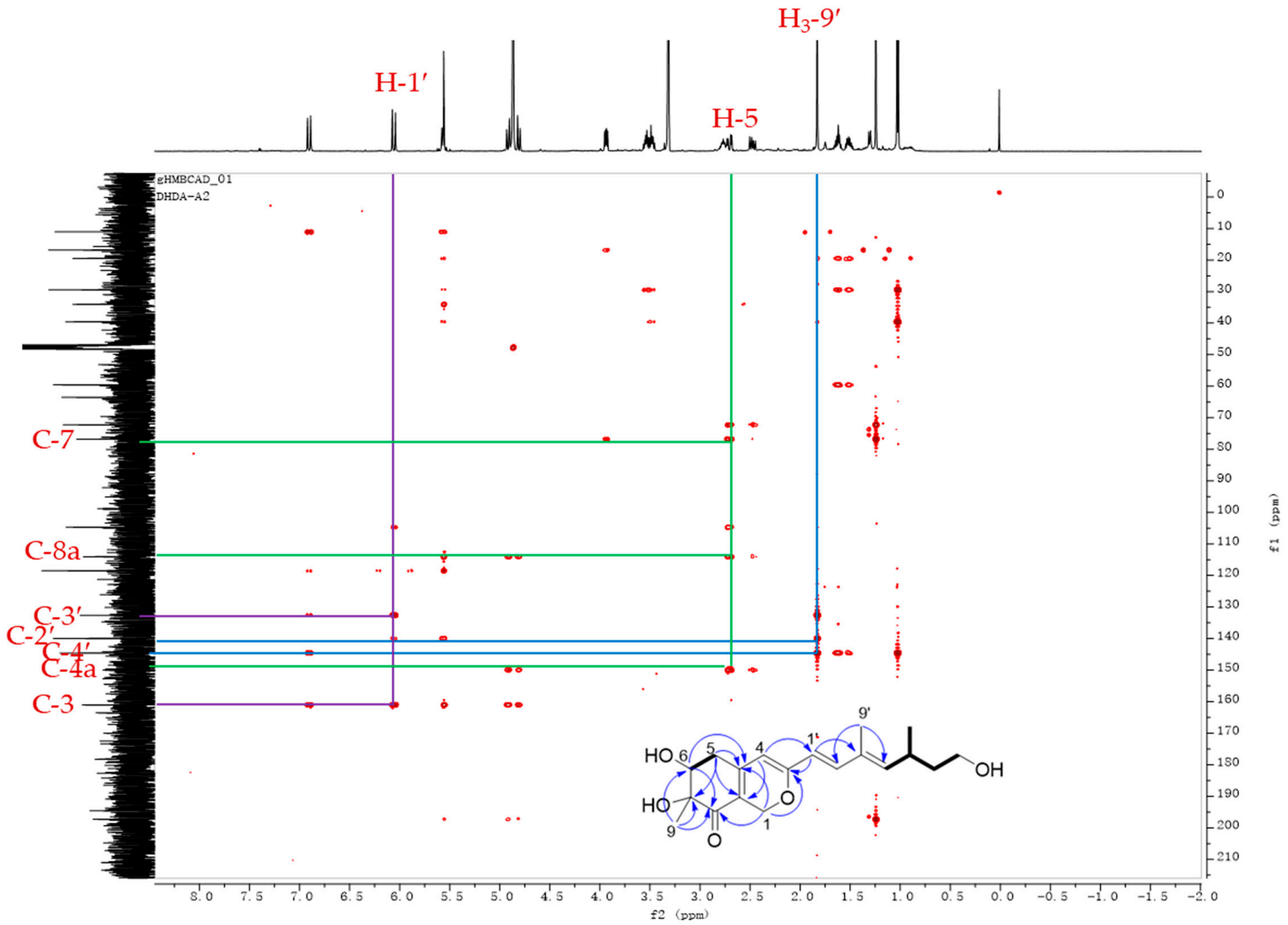

Figure S14. HMBC spectrum of compound **2** in methanol-d_4.

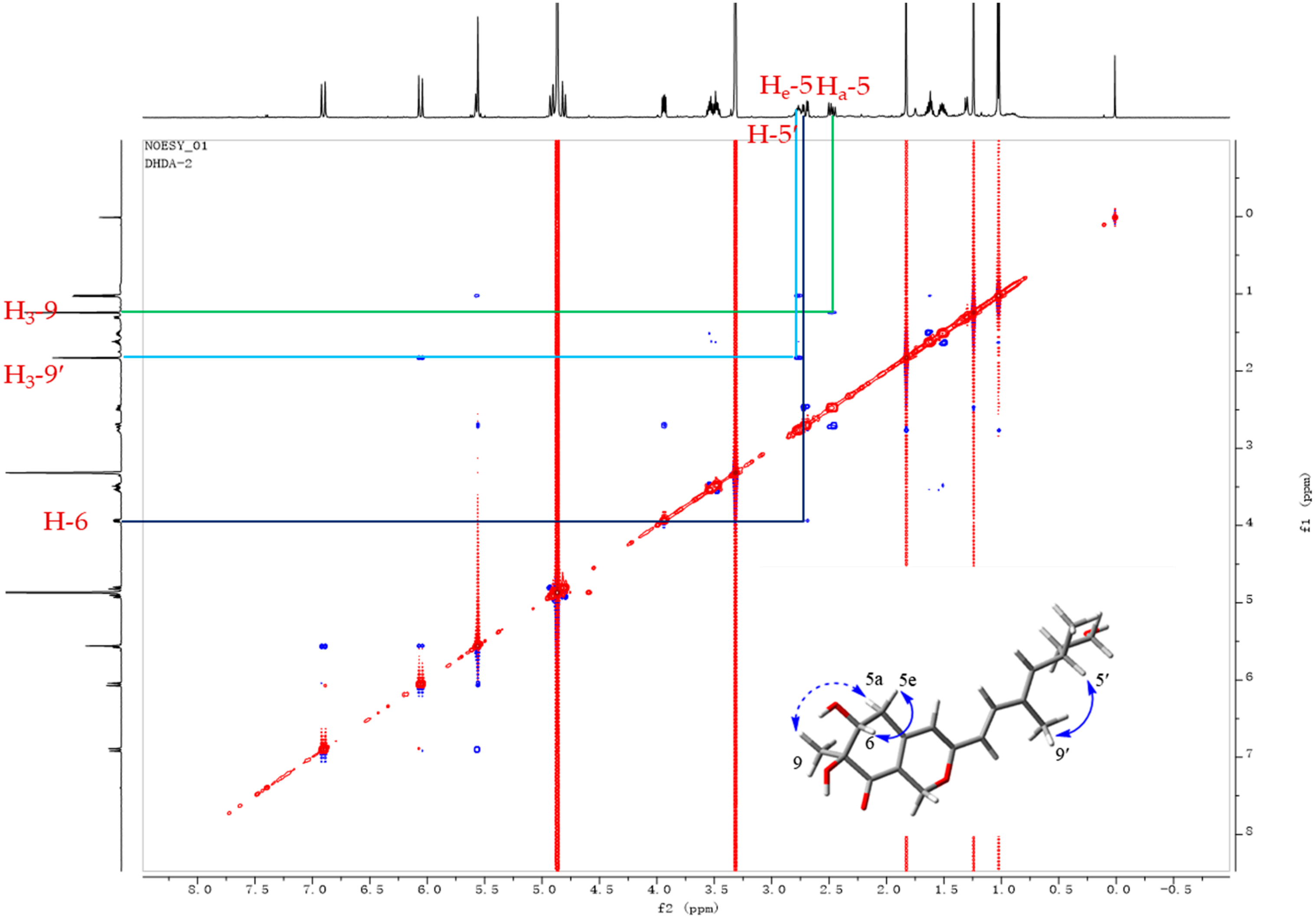

Figure S15. NOESY spectrum of compound **2** in methanol-d_4.

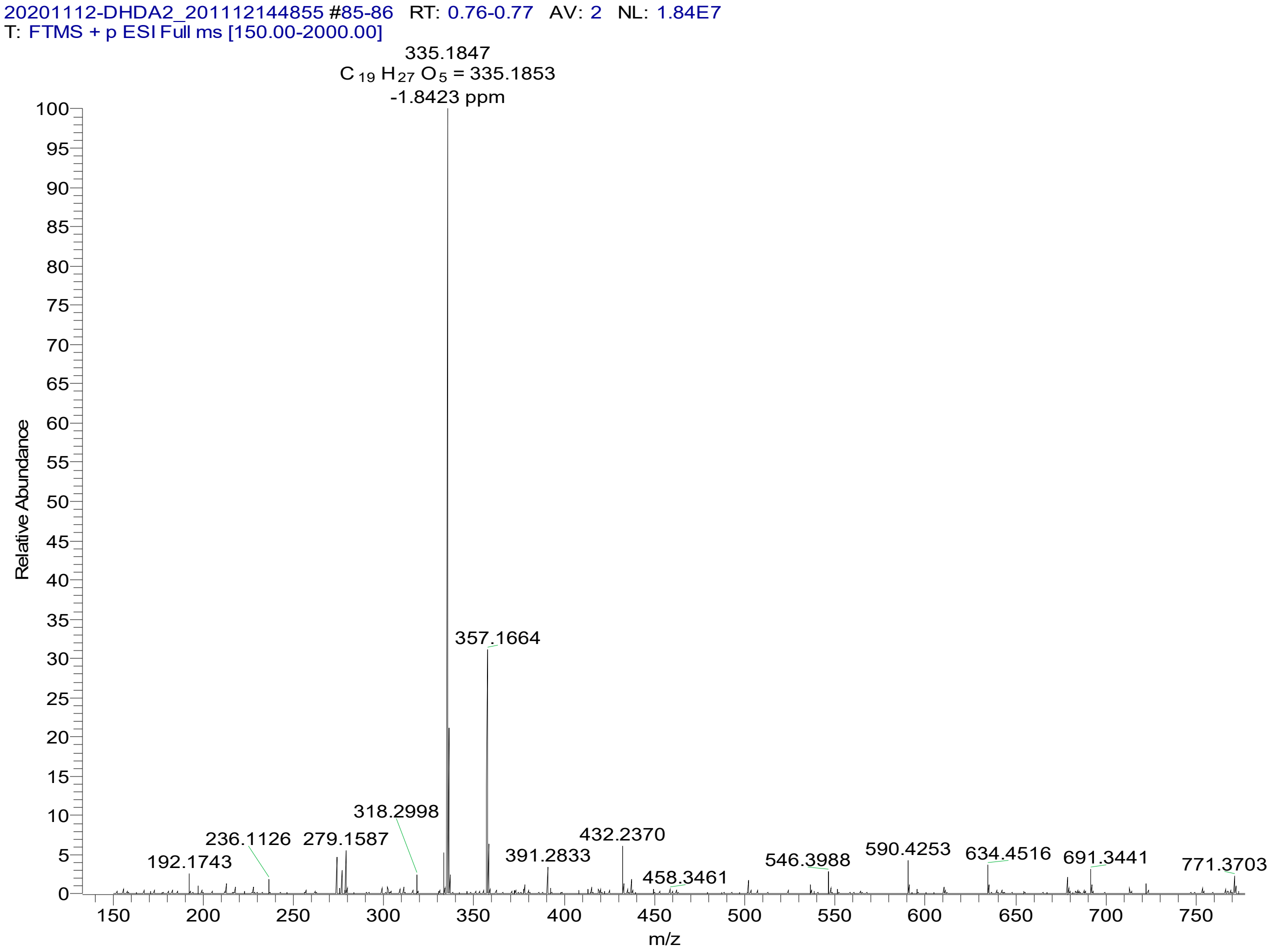

Figure S16. HRESIMS spectrum (positive ion mode) of compound **2**.

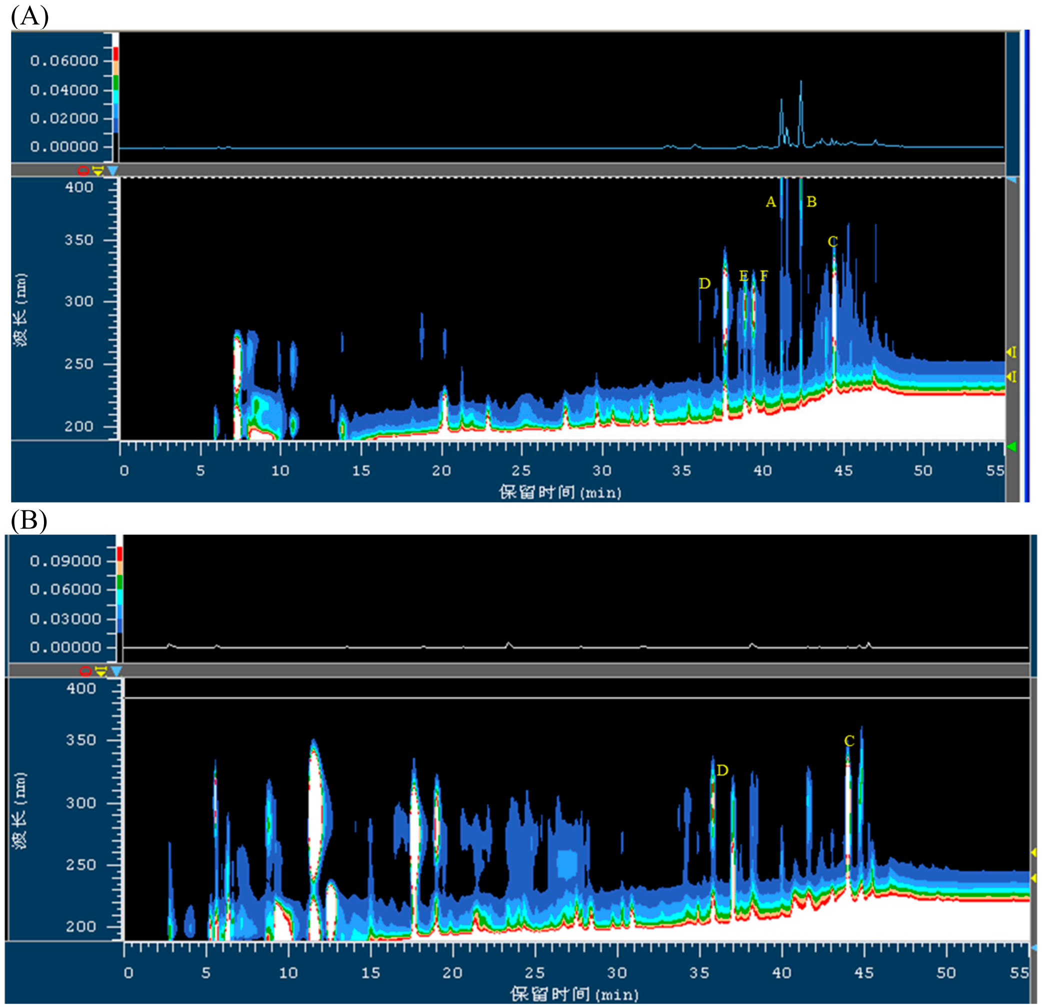

Figure S17. HPLC detection on ethyl acetate (EtOAc) extracts of Δ*hdaA* strain (A) and WT strain (B). (The compounds were marked with yellow letter, A: asperterilone A; B: asperterilone B; C: butyrolactone I; D: butyrolactone II; E: butyrolactone III; F: butyrolactone V.)

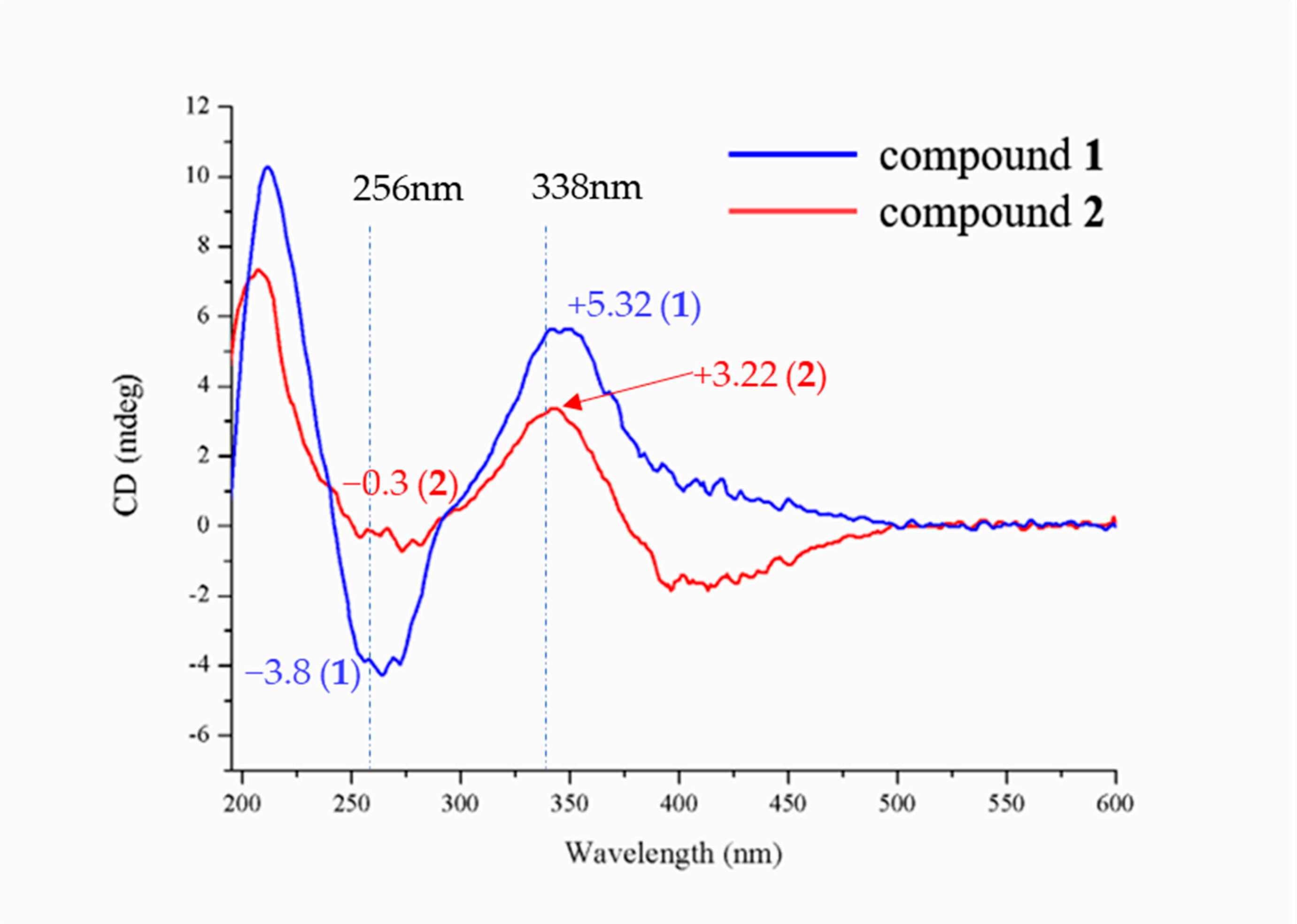

Figure S18. CD curves of compounds **1** and **2**. (The CD spectra of **1** and **2** showed positive [+5.32 (**1**), +3.22 (**2**) at 338nm] and negative [−3.80 (**1**), −0.30 (**2**) at 256nm] Cotton effects)

General PCR process

Pre-denaturation	95 °C	2 min	1cycle
Denaturation	95 °C	20 sec	30 cycles
Annealling	58 °C	20 sec	
Extension	72 °C	1 min	
Extension	72 °C	5 min	1cycle

Fusion PCR process

Pre-denaturation	95 °C	2 min	1cycle
Denaturation	95 °C	20 sec	12 cycles
Annealling	58 °C	10 min	
Extension	72 °C	5 min	
Extension	72 °C	5 min	1cycle

Figure S19. The PCR conditions for construction of mutant. (The homologous arms of *hdaA* (upstream and downstream) and *hph* marker gene were amplified through general PCR process, the *hdaA* deletion cassette with three DNA fragments was constructed by fusion PCR.).
